# Supplementary material for: Persistent symptoms and clinical findings in adults with post-acute sequelae of COVID-19/post-COVID-19 syndrome in the second year after acute infection: A population-based, nested case-control study
Source: PLoS Med. 2025 Jan 23;22(1):e1004511. doi: 10.1371/journal.pmed.1004511 (PMC12005676; doi:10.1371/journal.pmed.1004511)
Supplement: S1 STROBE Checklist — (PDF) [file pmed.1004511.s001.pdf]

STROBE Statement –
checklist of items that should be included in reports of observational studies

***Peter et al.* Persistent symptoms and clinical findings in adults with post-acute sequelae of COVID-19/post-COVID-19 syndrome in the second year after acute infection: population-based, nested case-control study.**

	Item No	Recommendation	Section, paragraph
**Title and abstract**	1	(*a*) Indicate the study's design with a commonly used term in the title or the abstract	See title
		(*b*) Provide in the abstract an informative and balanced summary of what was done and what was found	See abstract
**Introduction**			
Background/rationale	2	Explain the scientific background and rationale for the investigation being reported	Introduction, para 1-2
Objectives	3	State specific objectives, including any prespecified hypotheses	Introduction, para 3
**Methods**			
Study design	4	Present key elements of study design early in the paper	Methods – Study design and selection of participants, para 1
Setting	5	Describe the setting, locations, and relevant dates, including periods of recruitment, exposure, follow-up, and data collection	Methods – Study design and selection of participants, para 1-3
Participants	6	(*a*) *Cohort study*—Give the eligibility criteria, and the sources and methods of selection of participants. Describe methods of follow-up<br>*Case-control study*—Give the eligibility criteria, and the sources and methods of case ascertainment and control selection. Give the rationale for the choice of cases and controls<br>*Cross-sectional study*—Give the eligibility criteria, and the sources and methods of selection of participants	Methods – Study design and selection of participants, para 2 & 3
		(*b*) *Cohort study*—For matched studies, give matching criteria and number of exposed and unexposed<br>*Case-control study*—For matched studies, give matching criteria and the number of controls per case	Methods – Study design and selection of participants, para 3
Variables	7	Clearly define all outcomes, exposures, predictors, potential confounders, and effect modifiers. Give diagnostic criteria, if applicable	Methods – Data sources and measurements
Data sources/ measurement	8*	For each variable of interest, give sources of data and details of methods of assessment (measurement). Describe comparability of assessment methods if there is more than one group	Methods – Data sources and measurements & Supplementary text
Bias	9	Describe any efforts to address potential sources of bias	Methods – Statistical methods
Study size	10	Explain how the study size was arrived at	Methods – Study design and selection of participants, para 2 & 3, Flow-chart (Fig. 1)
Quantitative variables	11	Explain how quantitative variables were handled in the analyses. If applicable, describe which groupings were chosen and why	Methods – Statistical methods
Statistical methods	12	(*a*) Describe all statistical methods, including those used to control for confounding	Methods – Statistical methods

		(*b*) Describe any methods used to examine subgroups and interactions	Methods – Statistical methods
		(*c*) Explain how missing data were addressed	Methods – Statistical methods
		(*d*) *Cohort study*—If applicable, explain how loss to follow-up was addressed<br>*Case-control study*—If applicable, explain how matching of cases and controls was addressed<br>*Cross-sectional study*—If applicable, describe analytical methods taking account of sampling strategy	Methods – Statistical methods
		(*e*) Describe any sensitivity analyses	Results – Sensitivity analyses
**Results**			
Participants	13*	(a) Report numbers of individuals at each stage of study—eg numbers potentially eligible, examined for eligibility, confirmed eligible, included in the study, completing follow-up, and analysed	Flow-chart (Fig. 1)
		(b) Give reasons for non-participation at each stage	Flow-chart (Fig. 1)
		(c) Consider use of a flow diagram	Flow-chart (Fig. 1)
Descriptive data	14*	(a) Give characteristics of study participants (eg demographic, clinical, social) and information on exposures and potential confounders	Table 1
		(b) Indicate number of participants with missing data for each variable of interest	Indicated in each Fig. & Table
		(c) *Cohort study*—Summarise follow-up time (eg, average and total amount)	*NA*
Outcome data	15*	*Cohort study*—Report numbers of outcome events or summary measures over time	*NA*
		Case-control study—Report numbers in each exposure category, or summary measures of exposure	Table 1
		Cross-sectional study—Report numbers of outcome events or summary measures	*NA*
Main results	16	(*a*) Give unadjusted estimates and, if applicable, confounder-adjusted estimates and their precision (eg, 95% confidence interval). Make clear which confounders were adjusted for and why they were included	Fig. 3 & 4, Supplementary Figures/Tables
		(*b*) Report category boundaries when continuous variables were categorized	Table 1,2 & Supplementary tables S4 & S5
		(*c*) If relevant, consider translating estimates of relative risk into absolute risk for a meaningful time period	*NA*
Other analyses	17	Report other analyses done—eg analyses of subgroups and interactions, and sensitivity analyses	Results – Sensitivity analyses
**Discussion**			
Key results	18	Summarise key results with reference to study objectives	Discussion, para 1
Limitations	19	Discuss limitations of the study, taking into account sources of potential bias or imprecision. Discuss both direction and magnitude of any potential bias	Strengths and limitations, para 2
Interpretation	20	Give a cautious overall interpretation of results considering objectives, limitations, multiplicity of analyses, results from similar studies, and other relevant evidence	Discussion throughout
Generalisability	21	Discuss the generalisability (external validity) of the study results	Discussion – Strengths and limitations, para 2
**Other information**			
Funding	22	Give the source of funding and the role of the funders for the present study and, if applicable, for the original study on which the present article is based	See Funding
