# Supplementary material for: Persistent symptoms and clinical findings in adults with post-acute sequelae of COVID-19/post-COVID-19 syndrome in the second year after acute infection: A population-based, nested case-control study
Source: PLoS Med. 2025 Jan 23;22(1):e1004511. doi: 10.1371/journal.pmed.1004511 (PMC12005676; doi:10.1371/journal.pmed.1004511)
Supplement: S1 Appendix — (PDF) [file pmed.1004511.s002.pdf]

9618559890

Baden-Württembergische Long Covid-Studie

(EPILOC Phase 2)

Fragebogen für das ärztliche Gespräch

Angaben auf blau schraffiertem Hintergrund stammen aus der EPILOC Erstbefragung (Phase 1)

EPILOC Erstbefragung (Phase 1) vor etwa ___ Wochen

___ ___ Jahre

SARS-CoV-2 positiv ___ . ___

Abschnitt G: Fragen zum Gesundheitszustand

Ausfülldatum [Tag].[Monat].[Jahr] ☐☐.☐☐.☐☐☐☐

Haben Sie seit dem Sommer 2021 eine erneute Corona-Infektion (bestätigt durch PCR) durchgemacht? ja ☐ nein ☐

Falls *ja*, wann? **Testdatum** [Monat].[Jahr] ☐☐.☐☐☐☐

Um welche Virusvariante handelte es sich? unbekannt ☐ Delta ☐ Omikron ☐ sonstige ☐

Mussten Sie im Rahmen dieser Corona-Infektion ärztlich behandelt werden? ja ☐ nein ☐

Mussten Sie im Rahmen dieser Corona-Infektion im Krankenhaus stationär behandelt werden? ja ☐ nein ☐

Falls *ja*, wie lange? (einschließlich evtl. Liegezeiten auf einer Intensivstation) [Tage] ☐☐☐

Mussten Sie bei dieser Corona-Infektion intensivmedizinisch betreut werden? ja ☐ nein ☐

Falls *ja*, wie lange? (nur Liegezeiten auf einer Intensivstation) [Tage] ☐☐☐

Sie haben angegeben, dass Sie Ihren ursprünglichen Gesundheitszustand (vor Ihrem positiven Corona-Test) wie folgt wieder erreicht haben.

0%	10%	20%	30%	40%	50%	60%	70%	80%	90%	100%	100% entspricht vollständiger Erholung
☐	☐	☐	☐	☐	☐	☐	☐	☐	☐	☐	

Wie viel Prozent Ihres ursprünglichen Gesundheitszustands (vor Ihrem positiven Corona-Test) haben Sie heute wieder erreicht?

0%	10%	20%	30%	40%	50%	60%	70%	80%	90%	100%	100% entspricht vollständiger Erholung
☐	☐	☐	☐	☐	☐	☐	☐	☐	☐	☐	

8572559898

Nr.	Symptom	Ihre Angaben aus Phase 1: **Weiterhin** bestehend oder **neu** aufgetreten? ja	nein	Ihre aktuellen Angaben: **Weiterhin** bestehend oder **neu** aufgetreten? ja	nein (alle nein ☐)	Wie stark fühlen Sie sich dadurch **zurzeit** beeinträchtigt? Nicht	Leicht	Mäßig	Stark
1	Haarausfall	☐	☐	☐	☐	☐	☐	☐	☐
2	Kopfschmerzen	☐	☐	☐	☐	☐	☐	☐	☐
3	Bewusstseinsstörungen/Verwirrtheit	☐	☐	☐	☐	☐	☐	☐	☐
4	Konzentrationsstörungen	☐	☐	☐	☐	☐	☐	☐	☐
5	Gedächtnisstörungen	☐	☐	☐	☐	☐	☐	☐	☐
6	Angstzustände	☐	☐	☐	☐	☐	☐	☐	☐
7	depressive Verstimmung	☐	☐	☐	☐	☐	☐	☐	☐
8	Schwindel	☐	☐	☐	☐	☐	☐	☐	☐
9	Geruchsstörungen	☐	☐	☐	☐	☐	☐	☐	☐
10	Geschmacksstörungen	☐	☐	☐	☐	☐	☐	☐	☐
11	Husten	☐	☐	☐	☐	☐	☐	☐	☐
12	Heiserkeit	☐	☐	☐	☐	☐	☐	☐	☐
13	Halsschmerzen/-kratzen	☐	☐	☐	☐	☐	☐	☐	☐
14	pfeifende oder keuchende Atmung	☐	☐	☐	☐	☐	☐	☐	☐
15	Kurzatmigkeit	☐	☐	☐	☐	☐	☐	☐	☐
16	Brustschmerzen	☐	☐	☐	☐	☐	☐	☐	☐
17	Erbrechen	☐	☐	☐	☐	☐	☐	☐	☐

Sie hatten im Rahmen der damaligen akuten Corona-Infektion Beschwerden oder Symptome angegeben. Welche dieser Beschwerden bestehen aktuell und mit welchem Schweregrad?

5964559890

Nr.	Symptom	Ihre Angaben aus Phase 1: **Weiterhin** bestehend oder **neu** aufgetreten? ja	nein	Ihre aktuellen Angaben: **Weiterhin** bestehend oder **neu** aufgetreten? ja	nein	Wie stark fühlen Sie sich dadurch **zurzeit** beeinträchtigt? Nicht	Leicht	Mäßig	Stark
18	Übelkeit	☐	☐	☐	☐	☐	☐	☐	☐
19	Bauchschmerzen	☐	☐	☐	☐	☐	☐	☐	☐
20	Durchfall	☐	☐	☐	☐	☐	☐	☐	☐
21	Schüttelfrost	☐	☐	☐	☐	☐	☐	☐	☐
22	Fieber	☐	☐	☐	☐	☐	☐	☐	☐
23	Gliederschmerzen	☐	☐	☐	☐	☐	☐	☐	☐
24	Muskelschmerzen	☐	☐	☐	☐	☐	☐	☐	☐
25	Gelenkschmerzen	☐	☐	☐	☐	☐	☐	☐	☐
26	Missempfindungen, Kribbeln an Haut oder Gliedmaßen	☐	☐	☐	☐	☐	☐	☐	☐
27	Schlafstörungen	☐	☐	☐	☐	☐	☐	☐	☐
28	chronische Erschöpfung	☐	☐	☐	☐	☐	☐	☐	☐
29	Rasche körperliche Erschöpfung	☐	☐	☐	☐	☐	☐	☐	☐
30	Hautausschlag	☐	☐	☐	☐	☐	☐	☐	☐
31	andere Symptome	☐	☐	☐	☐	☐	☐	☐	☐
	was genau?								
	was genau?								

Ihre Angaben bei der ersten Befragung:
Haben Sie bereits eine (Erst-)Impfung gegen COVID-19 erhalten? ☐ ja ☐ nein

Falls *ja*, wann? [Monat].[Jahr] ☐☐.☐☐☐☐

mit? ☐ Comirnaty (BioNTech) ☐ Janssen (Johnson & Johnson) ☐ Spikevax (Moderna) ☐ Vaxzevria (AstraZeneca)

1. Falls Sie bei der ersten Befragung noch keine Impfung gegen COVID-19 erhalten hatten. Haben Sie inzwischen eine erste Impfung gegen COVID-19 erhalten?

☐ ja ☐ nein

Falls *ja*, wann? [Monat].[Jahr] ☐☐.☐☐☐☐

mit? ☐ Comirnaty (BioNTech) ☐ Janssen (Johnson & Johnson) ☐ Spikevax (Moderna) ☐ Vaxzevria (AstraZeneca) ☐ sonstige

2. Haben Sie inzwischen eine zweite Impfung gegen COVID-19 erhalten?

☐ ja ☐ nein

Falls *ja*, wann? [Monat].[Jahr] ☐☐.☐☐☐☐

mit? ☐ Comirnaty (BioNTech) ☐ Spikevax (Moderna) ☐ Vaxzevria (AstraZeneca) ☐ sonstige

3. Haben Sie inzwischen eine dritte Impfung gegen COVID-19 erhalten?

☐ ja ☐ nein

Falls *ja*, wann? [Monat].[Jahr] ☐☐.☐☐☐☐

mit? ☐ Comirnaty (BioNTech) ☐ Spikevax (Moderna) ☐ sonstige
