# Supplementary material for: Persistent symptoms and clinical findings in adults with post-acute sequelae of COVID-19/post-COVID-19 syndrome in the second year after acute infection: A population-based, nested case-control study
Source: PLoS Med. 2025 Jan 23;22(1):e1004511. doi: 10.1371/journal.pmed.1004511 (PMC12005676; doi:10.1371/journal.pmed.1004511)
Supplement: S2 Appendix — (PDF) [file pmed.1004511.s003.pdf]

S2 Appendix. Details of clinical assessments and validated questionnaires (with references)

mMRC. The mMRC dyspnoea scale assessment the disability due to breathlessness. It consists of five grades that contain of a description of different activities and moderately correlates with other healthcare-associated morbidity, mortality and quality of life scales (particularly in COPD). The instrument has commonly been used and recently recommended for COVID-19 and long COVID research.

- Mahler DA, Wells CK. Evaluation of clinical methods for rating dyspnea. *Chest* 1988; 93:580-6.
- Gorst SL, Seylanova N, Dodd SR, Harman NL, O'Hara M, Terwee CB, et al. Core outcome measurement instruments for use in clinical and research settings for adults with post-COVID-19 condition: an international Delphi consensus study. *Lancet Respir Med* 2023; 11:1101-14.

Handgrip strength test. The grip strength test measures the maximum isometric strength of the hand and forearm muscles. It uses a hydraulic dynamometer with the arm positioned at right angles and the elbow by the side of the body. The test subject squeezes the dynamometer with maximum isometric effort for about 5 seconds. Both hands are grip tested three times (in alternating manner), and the maximum strength is recorded. We used different devices, all CE marked and calibrated, including SAEHAN DHD-1, SH5001 and SH1003 hydraulic hand dynamometer devices (Saehan Corporation, Changwon-si, South Korea).

- Roberts HC, Denison HJ, Martin HJ, Patel HP, Syddall H, Cooper C, et al. A review of the measurement of grip strength in clinical and epidemiological studies: towards a standardised approach. *Age Ageing* 2011; 40:423-9.

Multifrequency bioelectrical impedance analysis. Whole body composition was measured using one of the following instruments: Inbody 770 (Biospace Korea, Seoul, South Korea), BIA 101 BIVA PRO (Akern s.r.l., Florence, Italy), InBody 4.0 (InBody Europe B.V., Eschborn, Deutschland). The measurements of the percentage of body fat was done according to standard procedures.

- Kyle UG, Bosaeus I, De Lorenzo AD, Deurenberg P, Elia M, Gómez JM, et al. Bioelectrical impedance analysis--part I: review of principles and methods. *Clin Nutr* 2004; 23:1226-43.

Validated questionnaires – health-related quality of life. We used the SF-12 Health Survey which is a short form of the SF-36 instrument to assess health-related quality of life. The 12 items are rated on a 5-point Likert scale and can be summarized as total score or evaluated on two subscales: the mental component and physical component summary. The German version has been tested in health individuals and other populations.

- Ware J, Kosinski M, Keller SD. A 12-Item Short-Form Health Survey: construction of scales and preliminary tests of reliability and validity. *Med Care* 1996; 34:220-33.
- Gandek B, Ware JE, Aaronson NK, Apolone G, Bjorner JB, Brazier JE, et al. Cross-validation of item selection and scoring for the SF-12 Health Survey in nine countries: results from the International Quality of Life Assessment project. *J Clin Epidemiol* 1998; 51:1171-8.
- Wirtz MA, Morfeld M, Glaesmer H, Brähler E. Normierung des SF-12 Version 2.0 zur Messung der gesundheitsbezogenen Lebensqualität in einer deutschen bevölkerungsrepräsentativen Stichprobe. *Diagnostica* 2018; 64:215-26.

Validated questionnaires – fatigue and sleep. We used the CFQ-11 for the assessment of the extent and severity of fatigue. The instrument is available in German language and has been tested in a representative sample of the German population. Each of the 11 items are answered on a 4-point Likert scale yielding a maximal global score of 33, or a maximal binary/bimodal score of 11. A bimodal score of >3 qualifies for "caseness", and a total score of >29 indicates extreme fatigue.

- Chalder T, Berelowitz G, Pawlikowska T, Watts L, Wessely S, Wright D, et al. Development of a fatigue scale. *J Psychosom Res* 1993; 37:147-53.

- Martin A, Staufenbiel T, Gaab J, Rief W, Brähler E. Messung chronischer Erschöpfung – teststatistische Prüfung der Fatigue Skala (FS). *Z Klin Psychol Psychother* 2010; 39:33-44.
- Cella M, Chalder T. Measuring fatigue in clinical and community settings. *J Psychosom Res* 2010; 69:17-22.

The PSQI was used to evaluate overall sleep quality (over the past month). The 19 items belong to one of seven subcategories: subjective sleep quality, sleep latency, sleep duration, habitual sleep efficiency, sleep disturbances, use of sleeping medication, and daytime dysfunction. A German version has been validated in healthy individuals and other populations.

- Buysse DJ, Reynolds CF, Charles F, Monk TH, Berman SR, Kupfer DJ. The Pittsburgh sleep quality index: a new instrument for psychiatric practice and research. *Psychiatry Research* 1989; 28:193-213.
- Backhaus J, Riemann D. Schlafstörungen bewältigen. Weinheim: Beltz Psychologie Verlags Union; 1996.

The ISI is a short 7-item screening instrument to assess the nature, severity, and impact of insomnia in the past two weeks. Its German version has been validated for the evaluation of the subjective perception of sleep complaints and compared with the PSQI in in healthy individuals and other populations. ISI scores range from 0 to 28 and can be interpreted as follows: <8, no insomnia, >14 insomnia (>21 severe insomnia). There was a high correlation between PSQI and ISI scores ($r=0.816$, $p<0.001$), but weaker correlations ($r<0.5$) between PSQI or ISI with ESS in the EPILOC phase 2 participant population.

- Bastien CH, Vallières A, Morin CM. Validation of the Insomnia Severity Index (ISI) as an outcome measure for insomnia research. *Sleep Med* 2001; 2:297-307.
- Morin CM, Belleville G, Bélanger L, Ivers H. The Insomnia Severity Index: psychometric indicators to detect insomnia cases and evaluate treatment response. *Sleep* 2011; 34:601-8.
- Gerber M, Lang C, Lemola S, Colledge F, Kalak N, Holsboer-Trachsler E, et al. Validation of the German version of the insomnia severity index in adolescents, young adults and adult workers: results from three cross-sectional studies. *BMC Psychiatry* 2016; 16:174.

The ESS was used to assess daytime sleepiness in middle-aged white individuals. Respondents rate their usual chances of dozing off or falling asleep in eight different daily situations on a 4-point scale. The total ESS score ranges between 0 and 24, with a higher score reflecting a higher level of daytime sleepiness. A score of <11 corresponds to the normal range of sleepiness in healthy adults. A validated German version of the ESS is available. In the EPILOC phase 2 participant population the correlation between ESS and PSQI and also ISI was moderate.

- Kendzerska TB, Smith PM, Brignardello-Petersen R, Leung RS, Tomlinson GA. Evaluation of the measurement properties of the Epworth sleepiness scale: a systematic review. *Sleep Med Rev* 2014; 18:321-31.
- Bloch KE, Schoch OD, Zhang JN, Russi EW. German version of the Epworth Sleepiness Scale. *Respiration* 1999; 66:440-7.
- Sander C, Hegerl U, Wirkner K, Walter N, Kocalevent RD, Petrowski K, et al. Normative values of the Epworth Sleepiness Scale (ESS), derived from a large German sample. *Sleep Breath* 2016; 20:1337-45.

Validated questionnaires – mood and anxiety. We used the 9-item PHQ-9 to screen for depressive symptom severity and the 7-item GAD-7 to assess anxiety. The PHQ-9 has strong diagnostic accuracy in assessing depression. Respondents are asked to rate each item on a 0 to 3 Likert-type scale how frequently they experienced each symptom over the past 2 weeks (0, not at all; 3, nearly every day). Scores are summed to yield a total score ranging from 0 to 27. Cut-off scores between 8 and 11 have good sensitivity and specificity for detecting depression, and values >14 indicate moderate to severe depression. A German version is available.

- Kroenke K, Spitzer RL, Williams JBW, Löwe B. The Patient Health Questionnaire Somatic, Anxiety, and Depressive Symptom Scales: a systematic review. *Gen Hosp Psychiatry* 2010;32:345–59.
- Manea L, Gilbody S, McMillan D. Optimal cut-off score for diagnosing depression with the Patient Health Questionnaire (PHQ-9): a meta-analysis. *CMAJ* 2012; 184:E191-6.

- Gräfe K, Zipfel S, Herzog W, Löwe B. Screening psychischer Störungen mit dem "Gesundheitsfragebogen für Patienten (PHQ-D)". Ergebnisse der deutschen Validierungsstudie. *Diagnostica* 2004; 50:171-81.

The GAD-7, originally developed to screen for generalized anxiety disorders in primary care settings, is now commonly used across various settings and populations. It has also been used to screen for post-traumatic stress disorder, social anxiety or panic disorders. The total score for the seven items ranges from 0 to 21. A score >9 indicates moderate to severe anxiety. A validated German version is available.

- Spitzer RL, Kroenke K, Williams JBW, Löwe B. A brief measure for assessing generalized anxiety disorder: the GAD-7. *Arch Intern Med* 2006; 166:1092-7.
- Löwe B, Müller S, Brähler E, Kroenke K, Albani C, Decker O. Validierung und Normierung eines kurzen Selbstratinginstrumentes zur Generalisierten Angst (GAD-7) in einer repräsentativen Stichprobe der deutschen Allgemeinbevölkerung. *Psychother Psychosom Med Psychol* 2007; 57:A050.

Validated questionnaires – perceived stress. The PSS-10 is a 10-item questionnaire developed to assess stress levels. It evaluates the degree to which an individual has perceived life as unpredictable, uncontrollable and overloading over the previous month, and captures perceived helplessness and self-efficacy. PSS-10 scores correlate with depression, anxiety and fatigue. A German version has been validated in healthy individuals and in patients with diverse mental illnesses.

- Cohen S, Kamarck T, Mermelstein R. A global measure of perceived stress. *J Health Soc Behav* 1983; 24:385-96.
- Klein EM, Brähler E, Dreier M, Reinecke L, Müller KW, Schmutzer G, et al. The German version of the Perceived Stress Scale - psychometric characteristics in a representative German community sample. *BMC Psychiatry* 2016; 16:159.
- Schneider EE, Schönfelder S, Domke-Wolf M, Wessa M. Measuring stress in clinical and nonclinical subjects using a German adaptation of the Perceived Stress Scale. *Int J Clin Health Psychol* 2020; 20:173-81.

Validated questionnaires – cognitive complaints. FLei is a questionnaire designed to assess subjective cognitive complaints. It is a 35-item instrument using a five-point rating scale (never; rarely; sometimes; often; very often) focusing on difficulties in everyday situations in the last 6 months in three areas (attention, memory and executive functioning). The subscores range between 0 and 40, and the total score ranges between 0 and 120, with higher scores indicating lower subjective cognitive ability. The FLei has been developed primarily for patients with mental disorders. It has earlier been used in patients with PCS in whom the mean total score was 69, and both the attention (mean, 25) and memory (mean, 26) subscores were higher than the executive subscore (mean 18). Also, there was a high correlation between the FLei total score and fatigue.

- Beblo T, Kunz M, Brokate B, Scheurich A, Weber B, Albert A, et al. Entwicklung eines Fragebogens zur subjektiven Einschätzung der geistigen Leistungsfähigkeit (FLei) bei Patienten mit psychischen Störungen. *Z Neuropsychol* 2010; 21:143-51.
- Delgado-Alonso C, Díez-Cirarda M, Pagán J, Pérez-Izquierdo C, Oliver-Mas S, Fernández-Romero L, et al. Unraveling brain fog in post-COVID syndrome: Relationship between subjective cognitive complaints and cognitive function, fatigue, and neuropsychiatric symptoms. *Eur J Neurol* 2023 Oct 5. doi: 10.1111/ene.16084.

Validated questionnaires – dysautonomia. We used the COMPASS-31 instrument to assess symptoms suspicious of autonomic nervous system dysfunction through six weighted symptom domains (orthostatic intolerance, vasomotor, secretomotor, gastrointestinal, bladder, pupillomotor. The questionnaire generates a weighted score from 0 to 100, with higher scores indicating worse autonomic dysfunction. A score of >19 suggests moderate-to-severe dysautonomia. In an earlier study with Long COVID patients, moderate-to-severe dysautonomia as measured by an increased COMPASS-31 score was frequent (66%), but not clearly linked to functional outcomes and quality of life. It has also been shown that the correlation between the COMPASS-31 score and instruments of objective measurement of dysautonomia is rather limited. A German version of the COMPASS-31 instrument has been validated.

- Sletten DM, Suarez GA, Low PA. COMPASS 31: A refined and abbreviated composite autonomic symptom score. *Mayo Clinic Proc* 2012; 87:1196-201.

- Larsen NW, Stiles LE, Shaik R, Schneider L, Muppidi S, Tsui CT, et al. Characterization of autonomic symptom burden in long COVID: a global survey of 2,314 adults. *Front Neurol* 2022; 13:1012668.
- Novak P, Systrom DM, Marciano SP, Knief A, Felsenstein D, Giannetti MP, et al. Mismatch between subjective and objective dysautonomia. *Sci Rep* 2024; 14:2513.
- Goldstein DS. Post-COVID dysautonomias: what we know and (mainly) what we don't know. *Nat Rev Neurol* 2024; 20:99-113.
- Hilz MJ, Wang R, Singer W. Validation of the Composite Autonomic Symptom Score 31 in the German language. *Neurol Sci* 2022; 43:365-71.

Cognitive tests. Cognitive status was assessed using the MoCA test, the SDMT and the TMT-B. In a subgroup of study participants (with a FLei memory subscore >19) we also performed the "Verbaler Lern- and Merkfähigkeitstest" (VLMT; Helmstaedter, Lendt & Lux: Verbaler Lern- und Merkfähigkeitstest. 1. Auflage 2001, Beltz Test, Göttingen) which is a German version of the "Auditory Verbal Learning Test" (AVLT; Lezak MD: Neuropsychological Assessment. 2nd Edition, 1983, Oxford University Press, New York). This test requires wordlist learning and allows the assessment of different memory parameters in one testing session. The results of this assessment will be analysed later and reported elsewhere.

The MoCA was validated as a sensitive tool for early detection of mild cognitive impairment. The instrument evaluates some cognitive functions including visuospatial ability, executive function, short-term and long-term memory recall, attention, language, abstraction, delayed recall, and orientation. The maximum score is 30 points. The original validation study suggested to use a cutoff of ≤26. Subsequent studies have shown that lower cutoff values (23 or 24) may yield fewer false-positive indications of cognitive impairment.

- Nasreddine ZS, Phillips NA, Bédirian V, Charbonneau S, Whitehead V, Collin I, et al. The Montreal Cognitive Assessment, MoCA: a brief screening tool for mild cognitive impairment. *J Am Geriatr Soc* 2005; 53:695-9.
- Thomann AE, Berres M, Goettel N, Steiner LA, Monsch AU. Enhanced diagnostic accuracy for neurocognitive disorders: a revised cut-off approach for the Montreal Cognitive Assessment. *Alzheimers Res Ther* 2020; 12:39.
- Malek-Ahmadi M, Nikkhahmanesh N. Meta-analysis of Montreal cognitive assessment diagnostic accuracy in amnestic mild cognitive impairment. *Front Psychol* 2024; 15:1369766.

The SDMT evaluates attention, visual scanning, motor speed, and associative learning. The test requires participants to match 9 abstract symbols paired with numerical digits. The scores are obtained after counting the correct number of associations within 90 seconds. It ranges from 0 to 110, with higher numbers indicating better performance. The test was performed as a written test according to the SDMT Manual (13th version, 2013). Normal scores vary by age, gender, and education.

- Smith A. Symbol Digits Modalities Test. Western Psychological Services, Los Angeles, 1982.
- Ryan J, et al. Normative data for the symbol digit modalities test in older white Australians and Americans, African-Americans, and Hispanic/latinos. *J Alzheimer's Dis Rep* 2020; 4:313-23.
- Fellows RP, Schmitter-Edgecombe M. Symbol digit modalities test: regression based normative data and clinical utility. Arch Clin Neuropsychol 2020; 35:105-15.

The TMT-B requires subjects to connect as quickly and correctly as possible 25 circles including both numbers and letters in an ascending pattern with the task of alternating between the numbers and letters. The amount of time required to complete the task reflects the respective score. The instrument captures mental tracking, motor speed, cognitive flexibility and selective attention. Mean scores in healthy individuals increase with age (50 s for 18-34 years; 75 s for 60-64 years) and depend on education levels.

- Reitan RM. Trail Making Test. Manual for administration and scoring. Tucson, AZ: Reitan Neuropsychology Laboratory, 1992.
- Gaudino EA, Geisler MW, Squires NK. Construct validity in the Trail Making Test: what makes Part B harder? *J Clin Exp Neuropsychol* 1995; 17:529-35.
- Tombaugh TN. Trail Making Test A and B: normative data stratified by age and education. *Arch Clin Neuropsychol* 2004; 19:203-14.
- Bowie CR, Harvey PD. Administration and interpretation of the trail making test. *Nat Protoc* 2006; 1:2277-81.
