# Supplementary material for: Persistent symptoms and clinical findings in adults with post-acute sequelae of COVID-19/post-COVID-19 syndrome in the second year after acute infection: A population-based, nested case-control study
Source: PLoS Med. 2025 Jan 23;22(1):e1004511. doi: 10.1371/journal.pmed.1004511 (PMC12005676; doi:10.1371/journal.pmed.1004511)
Supplement: S3 Appendix — (PDF) [file pmed.1004511.s004.pdf]

S3 Appendix. Methodological details of echocardiography and CPET (with references)

Echocardiography. Resting echocardiograms were performed with one of the following instruments: GE Vivid E9 (GE Healthcare, Solingen, Germany), EPIC7 (Philips Healthcare, Andovar, Massachusetts/USA), iE33 (Philips Medical Systems B.V., Eindhoven, Netherlands). Data sets were digitally stored and exported on a dedicated workstation. The assessment of biventricular and atrial size, LV diastolic function, LV and RV systolic function with evaluation of the LV-EF, LV-E/e' and LV-E/A was performed according to current guidelines. Images were uploaded and analysed centrally by two independent experienced examiners on TomTec Image Arena Version IA4.6.4, TTA2.20.01 (TomTec Imaging Systems GmbH, Unterschleißheim, Germany) as imaging analysis software.

- Lang RM, Badano LP, Mor-Avi V, Afilalo J, Armstrong A, Ernande L, et al. Recommendations for cardiac chamber quantification by echocardiography in adults: an update from the American Society of Echocardiography and the European Association of Cardiovascular Imaging. *Eur Heart J Cardiovasc Imaging* 2015; 16:233-70.
- Nagueh SF, Smiseth OA, Appleton CP, Byrd BF, Dokainish H, Edvardsen T, et al. Recommendations for the evaluation of left ventricular diastolic function by echocardiography: an update from the American Society of Echocardiography and the European Association of Cardiovascular Imaging. *Eur Heart J Cardiovasc Imaging* 2016; 17:1321-60.
- Gluckman TJ, Bhave NM, Allen LA, Chung EH, Spatz ES, Ammirati E, et al. 2022 ACC Expert Consensus Decision Pathway on cardiovascular sequelae of COVID-19 in adults: myocarditis and other myocardial involvement, post-acute sequelae of SARS-CoV-2 infection, and return to play: a report of the American College of Cardiology Solution Set Oversight Committee. J Am Coll Cardiol 2022; 79:1717-56.
- Wilson MG, Hull JH, Rogers J, Pollock N, Dodd M, Haines J, et al. Cardiorespiratory considerations for return-to-play in elite athletes after COVID-19 infection: a practical guide for sport and exercise medicine physicians. Br J Sports Med 2020; 54:1157-61.
- Szabó L, Juhász V, Dohy Z, Fogarasi C, Kovács A, Lakatos BK, et al. Is cardiac involvement prevalent in highly trained athletes after SARS-CoV-2 infection? A cardiac magnetic resonance study using sex-matched and age-matched controls. Br J Sports Med 2022; 56:553-60.

CPET. All CPETs were performed by means of stationary ergospirometers (Quark CPET, Rome, Italy; MetaLyzer, CORTEX Biophysics, Leipzig, Germany; or Ergostik, Geratherm, Geratal, Germany) using an electronically braked cycle ergometer. Patients received a standard explanation before the procedure and wore a non-rebreathing Hans-Rudolph mask connected to the respective ergospirometry system. CPET started with a 2 min resting phase followed by an unloaded pedalling phase of 2 min, a linear ramp increase in load tailored for each patient (continuous increase equivalent to an increase of 10, 15, 20, 25, 30, 35 or 40 W per minute) in order to achieve exhaustion in 6-12 min and a recovery phase of 3 min. The tests were done according to current recommendations as published by the German Respiratory Society.

During tests, pulmonary ventilation (VE), O_2 consumption (VO_2) and CO_2 output (VCO_2) were continuously measured breath-by-breath. Electrocardiogram (CardioPart 12, Amedtec, Aue, Germany; Custo med GmbH, Ottobrunn, Germany) and finger pulse oximetry (SpO2, Radical-7, Masimo, CA, USA; EDAN pulse oximeter H100B, Edan, San Diego, CA, USA) were continuously recorded. Blood pressure was measured manually. The level of dyspnoea and muscular fatigue was assessed at peak exercise using the Modified BORG Dyspnea Scale (Borg CR10), and the reason for exercise termination was recorded (fatigue, dyspnoea or undetermined). For objective determination of maximal effort, a respiratory exchange ratio (RER, VCO_2/VO_2) of >1.05 had been predetermined. VO_{2max} was determined as an average of the last 30 s period of exercise; percent of predicted for VO_{2max} was calculated according to Cooper and Storer, using the following formulas: male 50.02 – (0.384 X age) ml/kg/min; female 42.83 – (0.371 X age) ml/kg/min. Breathing reserve (BR) was continuously calculated as VE - (FEV1 x 40). The VE/VCO_2 slope was calculated over the linear component of

VE versus VCO_2. Values from 23 to 28 (males) and 26 to 30 (females), respectively, have been reported for normal healthy non-elderly adults.

- Meyer FJ, Borst MM, Buschmann HC, Claussen M, Dumitrescu D, Ewert R, et al. Belastungsuntersuchungen in der Pneumologie – Empfehlungen der Deutschen Gesellschaft für Pneumologie und Beatmungsmedizin e. V. *Pneumologie* 2018; 72:687-731.
- Borg G. Psychophysical scaling with applications in physical work and the perception of exercise. *Scand J Work Environ Health* 1990; 16 (Suppl 1): 55-8.
- Cooper B, Storer W. Exercise testing and interpretation. Cambridge University Press, Cambridge 2001.
- Mezzani A. Cardiopulmonary exercise testing: basics of methodology and measurements. Ann Am Thorac Soc 2017; 14(Suppl 1):S3-11.
