# Supplementary material for: Persistent symptoms and clinical findings in adults with post-acute sequelae of COVID-19/post-COVID-19 syndrome in the second year after acute infection: A population-based, nested case-control study
Source: PLoS Med. 2025 Jan 23;22(1):e1004511. doi: 10.1371/journal.pmed.1004511 (PMC12005676; doi:10.1371/journal.pmed.1004511)
Supplement: S4 Appendix — (PDF) [file pmed.1004511.s005.pdf]

S4 Appendix. Details of laboratory investigations

Biospecimen sampling and storage. On the day of the outpatient assessment and prior to further study procedures, we collected venous blood (a total of 88 mL), saliva and urine from non-fasting participants. Samples were either immediately analysed (see below) or stored below -20°C until further processing.

Blood counts and clinical chemistry. In brief, analyses included complete blood counts HbA1c (%), sodium (mmol/l), potassium (mmol/l), magnesium (mmol/l), creatinine (mg/dl), urea (mg/dl), estimated GFR (MDR and CKD-EPI G) (ml/min/1,73qm), LDH (U/l), CK (U/l), myoglobin (ng/ml), GPT/ALAT (U/l), GOT/ASAT (U/L), alcaline phosphatase (U/l), bilirubin (mg/dl), C-reactive protein or high sensitivity C-reactive protein (mg/l), TSH (mU/l), cortisol (nmol/l), ACTH (pg/ml), DHEA-S (µmol/l), Zn (µg/dl), Nt-proBNP (ng/l), ferritin (ng/ml), sTFR (mg/l), transferrin (g/l), IgE (mg/dl), 25-OH-vitamin D3 (ng/ml), CH50 (%), D-dimers (mg/l FEU), vWF antigen (%), vWF collagen binding activity (%), FVIII activity (%), urinary albumin, urinary creatinine. The assays were done at each site except for cortisol, ACTH and DHEA-S that were measured centrally using ECLIA tests on a Cobas (Roche) instrument, and CH50 that was measured only for study participants in Freiburg and Heidelberg.

Different instruments and kits (such as Immulite, Innovance, Cobas, Atellica, Advia) were used for some of the measurements depending on the analyser systems available on site. All analyses were performed on CE-marked calibrated instruments following the manufacturer's instructions. Reference values were obtained from each site for each analyte, and all results were analysed with adjustments for centre. There were no significant differences between patients with persistent PCS and recovered persons in the analytes not shown in the main text.

SARS-CoV-2 serology. For serological analyses, blood samples were sent to the respective diagnostic laboratories, and serum was prepared on the same day. Samples were either immediately analysed or stored at -20°C until further processing.

Antibodies against SARS-CoV-2 S1 receptor binding domain of the viral spike glycoprotein and nucleocapsid (N) were analysed locally. Antibodies against the SARS-CoV-2 N protein were measured using the Elecsys Anti-SARS-CoV-2 IgG/IgM ECLIA test kit (Roche Diagnostics) using a Roche Cobas e610 or e411 module (Heidelberg, Tübingen and Ulm) or by recomWell SARS-CoV-2 IgG ELISA (Mikrogen Diagnostik) run on a Siemens BEP III analyzer (Freiburg). Samples were analyzed by CLIA for IgG reactive to S1 with sCOVG ELISA Assays (Siemens Healthineers) using a Siemens ADVIA centaur instrument (Freiburg, Heidelberg, Tübingen) or with the Elecsys Anti-SARS-CoV-2 S test kit (Roche Diagnostics) on a Roche Cobas e402 instrument (Ulm). Data from FR, TÜ and UL were reported qualitatively. For samples collected in HD, diluted sera were analysed to obtain quantitative values (BAU/ml). All analyses were performed following the manufacturer's instructions.

EBV and CMV serology. Quantitative measurements of herpes virus specific antibodies were performed centrally in HD. Samples were frozen and shipped for analysis. Antibodies against Epstein-Barr-Virus antigens (IgG and IgM against VCA, IgG against EBNA, IgG against EA-D) were quantified using Enzyme-Linked Immunosorbent Assays (ELISA) (Euroimmun, Lübeck, Germany; EI 2793-9601 G, EI 2795 G) using an Euroimmune Analyzer II. IgG and IgM antibodies against Cytomegalovirus (CMV) were measured by ELISA (Euroimmun; EI 2570-9601 G,) on an Euroimmune Analyzer II ELISA values <16 relative units/ml (RE/ml) were classified as negative, 16-<22 as borderline and >=22 as positive. IgM antibodies against CMV were determined by ELISA using the CMV-IgM-ELA assay PKS (Medac, Hamburg, Germany) and a BEPIII analyzer (Siemens Healthineers). Samples with a ratio of >1.1 were classified as positive. All measurements were

performed according to the manufacturer's instructions. Due to few cases with IgM antibodies (EBV-VCA, 2 study participants; CMV, 11 study participants) these results were not further evaluated for differences between groups.

SARS-CoV-2 antigen measurements in plasma. Plasma samples from a subset of the cohort (100 individuals with persistent PCS and 100 recovered individuals) were analysed for traces of spike antigen using an ultrasensitive antigen ECL assay (S-PLEX SARS-CoV2 spike kit, K150ADJS), Mesoscale Discoveries, USA) on a Meso QuickPlex Q 60MM instrument, according to the manufacturer's instructions. The sensitivity of this method given by the manufacturer was 95 fg/ml; the dynamic range in our measurements was ~1-1000 pg/ml.

SARS-CoV-2 RT-PCR in faecal samples. Stool samples were collected by the patients at home in faecal sample collection media (9 ml of DNA-stabilisation solution R1100-250, Zymo DNA/RNA shield fecal collection tube R1101-E, Zymo research corporation; sample stability of four weeks at room temperature ensured by the manufacturer) shortly before their appointment for the clinical examination. Stool samples provided by the participants (from Heidelberg) were directly processed for RT-PCR analysis to include samples from 156 participants with persistent PCS and 103 participants with continued recovery (that were all negative).

RNA extraction from stool samples was carried out by a semi-automatical spin-column preparation using QIAamp Viral RNA Mini Kit (Qiagen, Germany) and Qiagen Qiacube according to the manufacturer's instructions. Isolated RNA was stored at -80°C.140 µL of purified RNA were used as template for real-time RT-PCR performed on a (Light cycler 480 II; Roche) using the LightMix Modular SARS-CE assay (#50-0776-96, TIB MolBiol, Berlin, Germany). Samples were classified as positive if the cycle threshold (Ct) value was ≤ 40. The housekeeping gene ß-actin served as an internal control to validate RNA isolation and reverse transcription efficiency.
