# Supplementary material for: Persistent symptoms and clinical findings in adults with post-acute sequelae of COVID-19/post-COVID-19 syndrome in the second year after acute infection: A population-based, nested case-control study
Source: PLoS Med. 2025 Jan 23;22(1):e1004511. doi: 10.1371/journal.pmed.1004511 (PMC12005676; doi:10.1371/journal.pmed.1004511)
Supplement: S1 Table — (PDF) [file pmed.1004511.s006.pdf]

S1 Table. Characteristics of participants with PCS and with recovery from phase 1 who participated in the phase 2 clinical examination.

	Participants with PCS		Participants with recovery	
	N	Mean or frequency	N	Mean or frequency
Phase 1 characteristics				
Female, N (%)		633 (64.5)		379 (65.8)
Age (years), mean (sd)	982	48.1 (12.3)	576	48.5 (12.3)
University entrance qualification, N (%)	982	420 (42.8)	576	338 (58.7)
Married/living together	977	847 (86.7)	574	509 (88.7)
Never Smoker, N (%)	979	629 (63.7)	576	420 (73.0)
Obese (≥30 kg/m²), N (%)		225 (23.0)		58 (10.1)
Full-time employment, N (%)	981	500 (51.0)	575	289 (50.3)
Treatment of acute SARS-CoV-2 infection, N (%)				
No medical care/treatment	968	541 (55.9)	573	516 (90.1)
Outpatient care		350 (36.2)		49 (8.6)
Inpatient care (without ICU)		62 (6.4)		6 (1.1)
Intensive care		15 (1.6)		2 (0.4)
Time from positive PCR test to phase 1 (months), mean (sd)	980	8.4 (1.6)	571	8.6 (1.6)
Received first SARS-CoV-2 vaccine, N (%) prior to phase 1	981	833 (84.9)	575	501 (87.1)
Preexisting condition/comorbidities, N (%)				
Musculoskeletal disorders (including rheumatism)	969	429 (44.3)	575	170 (29.6)
Cardiovascular disorders (including hypertension)	974	223 (22.9)	576	73 (12.7)
Neurological or sensory disorders	978	225 (23.0)	576	90 (15.6)
Metabolic disorders	981	230 (23.5)	575	88 (15.3)
Mental disorders	976	185 (19.0)	575	39 (6.8)
Respiratory diseases	975	163 (16.7)	576	48 (8.3)
Dermatological diseases	977	130 (13.3)	574	65 (11.3)
Cancer	957	39 (4.1)	560	26 (4.6)
History from phase 1 to phase 2				
Time from phase 1 to phase 2 (months), mean (sd)	982	9.1 (2.6)	576	8.4 (2.7)
Secondary SARS-CoV-2 infection since phase 1, N (%)	982	230 (23.4)	576	134 (23.3)
Total number of vaccine doses received, N (%)				
0	981	33 (3.4)	577	33 (5.7)
1		106 (10.8)		39 (6.8)
2		752 (76.7)		446 (77.3)
3		90 (9.2)		59 (10.2)
Participation in post-COVID-rehabilitation program, N (%)	941	90 (9.6)	565	0 (0.0)
Physician consultations within six months prior to phase 2, N (%)				
None	982	265 (27.0)	576	335 (58.2)
General practitioner		341 (34.7)		193 (33.5)
Any specialist physician		469 (47.8)		56 (9.7)
Cardiology		178 (18.1)		15 (2.6)
Respiratory Medicine		214 (21.8)		18 (3.1)
Neurology		88 (9.0)		1 (0.2)
Radiology		85 (8.7)		8 (1.4)
Rheumatology		15 (1.5)		2 (0.4)
Otorhinolaryngology		44 (4.5)		2 (0.4)
