# Supplementary material for: Persistent symptoms and clinical findings in adults with post-acute sequelae of COVID-19/post-COVID-19 syndrome in the second year after acute infection: A population-based, nested case-control study
Source: PLoS Med. 2025 Jan 23;22(1):e1004511. doi: 10.1371/journal.pmed.1004511 (PMC12005676; doi:10.1371/journal.pmed.1004511)
Supplement: S2 Table — (PDF) [file pmed.1004511.s007.pdf]

S2 Table. Raw and mutually adjusted predictors of case-control status change between phase 1 and phase 2.

Phase 1 characteristics/variable	Persistent PCS (N=613)	PCS with improvement (N=297)	
**Participants with PCS *(N=953)***	**OR (95%-CI)**	**OR (95%-CI)**	**OR_{adj} (95%-CI)**
Female sex	1.00	0.84 (0.63 to 1.10)	1.15 (0.82 to 1.60)
Age (per 10 years)	1.00	0.84 (0.76 to 0.94)	0.92 (0.80 to 1.04)
University entrance qualification	1.00	1.67 (1.27 to 2.18)	1.38 (1.01 to 1.89)
Not married/living together	1.00	1.07 (0.79 to 1.44)	0.90 (0.64 to 1.28)
Full-time employment	1.00	1.83 (1.39 to 2.40)	1.93 (1.39 to 2.67)
Medical care/treatment of acute infection	1.00	0.61 (0.46 to 0.81)	0.68 (0.51 to 0.92)
Obesity (BMI ≥ 30kg/m²)	1.00	0.61 (0.43 to 0.85)	0.73 (0.51 to 1.06)
Time from phase 1 to phase 2 (per month)	1.00	1.00 (0.95 to 1.06)	1.01 (0.96 to 1.07)
Secondary SARS-CoV-2 infection since Ph1	1.00	1.10 (0.80 to 1.50)	1.01 (0.71 to 1.44)
Two or more vaccine shots received	1.00	1.00 (0.68 to 1.46)	0.87 (0.58 to 1.32)
Participation in a post-COVID-19-rehabilitation program	1.00	0.26 (0.14 to 0.49)	0.41 (0.21 to 0.79)
Any specialist consultation in the last 6 months	1.00	0.49 (0.37 to 0.64)	0.60 (0.45 to 0.81)
	**Continued recovery (N=445)**	**Recovery with worsening health (N=123)**	
**Participants with recovery *(N=573)***	**OR (95%-CI)**	**OR (95%-CI)**	**OR_{adj} (95%-CI)**
Female sex	1.00	0.93 (0.61 to 1.41)	1.01 (0.62 to 1.62)
Age (per 10 years)	1.00	1.00 (0.85 to 1.17)	0.96 (0.79 to 1.17)
University entrance qualification	1.00	0.59 (0.39 to 0.88)	0.53 (0.34 to 0.83)
Not married/living together	1.00	1.23 (0.77 to 1.96)	1.26 (0.73 to 2.15)
Full-time employment	1.00	1.16 (0.78 to 1.73)	1.13 (0.72 to 1.79)
Medical care/treatment of acute infection	1.00	1.39 (0.74 to 2.60)	1.31 (0.69 to 2.51)
Obesity	1.00	1.58 (0.86 to 2.89)	1.62 (0.85 to 3.07)
Time from phase 1 to phase2 (per month)	1.00	0.99 (0.92 to 1.07)	0.94 (0.86 to 1.02)
Secondary SARS-CoV-2 infection since Ph1	1.00	1.72 (1.11 to 2.68)	1.85 (1.12 to 3.06)
Two or more vaccine shots received	1.00	1.05 (0.57 to 1.92)	1.19 (0.62 to 2.29)
