# Supplementary material for: Persistent symptoms and clinical findings in adults with post-acute sequelae of COVID-19/post-COVID-19 syndrome in the second year after acute infection: A population-based, nested case-control study
Source: PLoS Med. 2025 Jan 23;22(1):e1004511. doi: 10.1371/journal.pmed.1004511 (PMC12005676; doi:10.1371/journal.pmed.1004511)
Supplement: S3 Table — (PDF) [file pmed.1004511.s008.pdf]

S3 Table. Current medication by case-control status as reported at clinical examination in phase 2.

	Persistent PCS		PCS with improvement		Recovery with worsening health		Continued recovery	
	N	Mean or frequency	N	Mean or frequency	N	Mean or frequency	N	Mean or frequency
Current medication - number of different drugs, N (%)	664		318		124		452	
None		191 (28.8)		128 (40.3)		53 (42.7)		239 (52.9)
More than two		229 (34.5)		60 (18.9)		18 (14.5)		55 (12.2)
Current medication - ATC-Groups, N (%)	664		318		124		452	
Agents acting on the renin-angiotensin system (C09)		134 (20.2)		58 (18.2)		22 (17.7)		51 (11.3)
Thyroid therapy (H03)		136 (20.5)		50 (15.7)		14 (11.3)		63 (13.9)
Vitamins (A11)		106 (16.0)		25 (7.9)		12 (9.7)		31 (6.9)
Vitamin D (A11CC)		91 (13.7)		18 (5.7)		9 (7.3)		28 (6.2)
Drugs for obstructive airway diseases (R03)		75 (11.3)		29 (9.1)		8 (6.5)		15 (3.3)
Lipid modifying agents (C10)		71 (10.7)		16 (5.0)		5 (4.0)		31 (6.9)
Psychoanaleptics (N06)		78 (11.8)		15 (4.7)		7 (5.7)		9 (2.0)
Beta blocking agents (C07)		71 (10.7)		13 (4.1)		3 (2.4)		14 (3.1)
Analgesics (N02)		61 (9.2)		14 (4.4)		8 (6.5)		15 (3.3)
Drugs for peptic ulcer and gastro-oesophageal reflux disease (A02B)		59 (8.9)		18 (5.7)		4 (3.2)		8 (1.8)
Calcium channel blockers (C08)		41 (6.2)		10 (3.1)		3 (2.4)		19 (4.2)
Diuretics (C03)		37 (5.6)		10 (3.1)		2 (1.6)		12 (2.7)
Antithrombotic agents (B01)		38 (5.7)		6 (1.9)		1 (0.8)		12 (2.7)
Drugs used in diabetes (A10)		31 (4.7)		8 (2.5)		3 (2.4)		5 (1.1)
Antihistamines for systemic use (R06)		22 (3.3)		9 (2.8)		5 (4.0)		8 (1.8)
Antiinflammatory and antirheumatic products (M01)		20 (3.0)		5 (1.6)		2 (1.6)		9 (2.0)
