# Supplementary material for: Persistent symptoms and clinical findings in adults with post-acute sequelae of COVID-19/post-COVID-19 syndrome in the second year after acute infection: A population-based, nested case-control study
Source: PLoS Med. 2025 Jan 23;22(1):e1004511. doi: 10.1371/journal.pmed.1004511 (PMC12005676; doi:10.1371/journal.pmed.1004511)
Supplement: S4 Table — (PDF) [file pmed.1004511.s009.pdf]

S4 Table. Additional results of resting heart ultrasound examination and CPET analyses by case-control status as reported at clinical examination in phase 2.

	Persistent PCS				PCS with improvement				Recovery with worsening health				Continued recovery			
	N	Frequency	OR (95%-CI)[1]	OR_{adj} (95%-CI)[2]	N	Frequency	OR (95%-CI)[1]	OR_{adj}[2]	N	Frequency	OR (95%-CI)[1]	OR_{adj} (95%-CI)[2]	N	Frequency	OR (95%-CI)[1]	OR_{adj} (95%-CI)[2]
Diastolic dysfunction grade 1/2	608	188 (30.9)	1.57 (1.16 to 2.11)	0.93 (0.67 to 1.30)	294	69 (23.5)	1.14 (0.79 to 1.66)	0.87 (0.59 to 1.29)	115	34 (29.6)	1.34 (0.83 to 2.16)	1.11 (0.67 to 1.84)	424	93 (21.9)	1.00 (ref.)	1.00 (ref.)
FEV1/FVC <70% of predicted	551	57 (10.3)	1.12 (0.72 to 1.74)	1.08 (0.68 to 1.71)	273	23 (8.4)	0.82 (0.47 to 1.41)	0.78 (0.68 to 1.71)	105	11 (10.5)	1.15 (0.56 to 2.37)	1.01 (0.49 to 2.10)	384	37 (9.6)	1.00 (ref.)	1.00 (ref.)
VE/VCO_2 slope																
>30		194 (34.9)	2.57 (1.87 to 3.52)	2.28 (1.64 to 3.17)		84 (30.1)	1.87 (1.29 to 2.70)	1.78 (1.22 to 2.58)		24 (22.9)	1.40 (0.82 to 2.39)	1.25 (0.73 to 2.15)		73 (18.5)	1.00 (ref.)	1.00 (ref.)
>34		75 (13.5)	3.86 (2.21 to 6.77)	2.98 (1.67 to 5.32)		26 (9.3)	2.41 (1.26 to 4.60)	2.14 (1.11 to 4.12)		6 (5.7)	1.48 (0.56 to 3.91)	1.19 (0.44 to 3.18)		16 (4.1)	1.00 (ref.)	1.00 (ref.)
VO_{2max} (ml/min/kg)	556				279				105				394			
<35 in men or <27 in women		405 (72.8)	3.89 (2.93 to 5.16)	2.43 (1.74 to 3.39)		168 (60.2)	2.28 (1.65 to 3.15)	1.92 (1.32 to 2.81)		63 (60.0)	2.11 (1.34 to 3.31)	1.80 (1.07 to 3.06)		165 (41.9)	1.00 (ref.)	1.00 (ref.)
<20 in men or <17 in women		69 (12.4)	18.6 (5.81 to 59.8)	9.44 (2.86 to 31.1)		12 (4.3)	6.23 (1.74 to 22.4)	4.34 (1.17 to 16.1)		2 (1.9)	2.62 (0.43 to 16.0)	2.02 (0.32 to 12.8)		3 (0.8)	1.00 (ref.)	1.00 (ref.)
VO_{2max} <85% of predicted		196 (35.3)	6.51 (4.34 to 9.77)	5.16 (3.29 to 8.07)		63 (22.6)	3.50 (2.20 to 5.57)	3.12 (1.88 to 5.20)		13 (12.4)	1.61 (0.81 to 3.22)	1.41 (0.67 to 2.98)		33 (8.4)	1.00 (ref.)	1.00 (ref.)

[1] Adjusted for sex-age class combinations, study centre only

[2] additionally adjusted for university entrance qualification, smoking status and use of beta blocking agents.

FEV1/FVC: Forced Expiratory Volume in 1 second divided by Forced Vital Capacity (a.k.a. Tiffeneau-Index)

VE/VCO_2 slope: Ventilation (VE) increases in relation to Carbon Dioxide production (VCO_2) during physical exertion

VO_{2max}: Maximal oxygen uptake
