# Supplementary material for: Persistent symptoms and clinical findings in adults with post-acute sequelae of COVID-19/post-COVID-19 syndrome in the second year after acute infection: A population-based, nested case-control study
Source: PLoS Med. 2025 Jan 23;22(1):e1004511. doi: 10.1371/journal.pmed.1004511 (PMC12005676; doi:10.1371/journal.pmed.1004511)
Supplement: S5 Table — (PDF) [file pmed.1004511.s010.pdf]

S5 Table. Neurocognitive tests by CPET results in participants with persistent PCS reported at clinical examination in phase 2.

	MoCA <25				SDMT <36[1]				TMT-B >109 sec[2]			
	N	N (%)	OR (95%-CI)[3]	OR (95%-CI)[4]	N	< 36[1], N (%)	OR (95%-CI)[3]	OR (95%-CI)[4]	N	N (%)	OR (95%-CI)[3]	OR (95%-CI)[4]
FEV1/FVC												
≥ 70%	493	164 (33.3)	1.00	1.00	493	63 (12.8)	1.00	1.00	493	71 (14.4)	1.00	1.00
< 70%	56	20 (35.7)	0.85 (0.45 to 1.58)	0.79 (0.41 to 1.53)	55	7 (12.7)	0.94 (0.38 to 2.32)	0.72 (0.27 to 1.90)	56	7 (12.5)	0.75 (0.31 to 1.84)	0.65 (0.26 to 1.66)
VE/VCO_2 slope												
≤ 30	360	104 (28.9)	1.00	1.00	359	43 (12.0)	1.00	1.00	360	48 (13.3)	1.00	1.00
> 30	194	81 (41.8)	1.50 (1.02 to 2.21)	1.53 (1.03 to 2.28)	194	27 (13.9)	1.13 (0.66 to 1.94)	1.08 (0.62 to 1.90)	194	31 (16.0)	1.09 (0.65 to 1.84)	1.02 (0.60 to 1.74)
> 34	75	40 (53.3)	2.38 (1.40 to 4.04)	2.06 (1.19 to 3.57)	75	14 (18.7)	2.16 (1.07 to 4.37)	1.80 (0.86 to 3.74)	75	12 (16.0)	1.04 (0.50 to 2.16)	0.88 (0.42 to 1.87)
VO_2 max, N (%)												
≥ 85% of predicted	358	120 (33.5)	1.00	1.00	358	43 (12.0)	1.00	1.00	358	54 (15.1)	1.00	1.00
< 85% of predicted	196	65 (33.2)	1.18 (0.79 to 1.78)	1.03 (0.63 to 1.69)	195	27 (13.9)	1.94 (1.08 to 3.49)	2.36 (1.14 to 4.86)	196	25 (12.8)	1.17 (0.66 to 2.06)	1.13 (0.58 to 2.22)

[1] 15%-percentile of stable controls

[2] 85%-percentile of stable controls

[3] controlled for sex-age class combinations and study centre

[4] additionally adjusted for university entrance qualification, BMI, smoking status, and use of beta blocking agents

FEV1/FVC: Forced Expiratory Volume in 1 second divided by Forced Vital Capacity (a.k.a. Tiffeneau-Index)

VE/VCO_2 slope: Ventilation (VE) increases in relation to Carbon Dioxide production (VCO_2) during physical exertion

VO_{2max}: Maximal oxygen uptake

MoCA: Montreal cognitive assessment

SDMT: Symbol Digit Modalities Test

TMT-B: Trail Making Test Part B
