# Supplementary material for: Persistent symptoms and clinical findings in adults with post-acute sequelae of COVID-19/post-COVID-19 syndrome in the second year after acute infection: A population-based, nested case-control study
Source: PLoS Med. 2025 Jan 23;22(1):e1004511. doi: 10.1371/journal.pmed.1004511 (PMC12005676; doi:10.1371/journal.pmed.1004511)
Supplement: S6 Table — (PDF) [file pmed.1004511.s011.pdf]

S6 Table. Case-control status by D-dimer levels (normal vs elevated).

	D-dimer		OR (95%-CI)[1]	OR_{adj} (95%-CI)[2]
	≤0.25 mg/L FEU	>0.25 mg/L FEU		
	N (%)	N (%)		
Continued recovery	279 (62.1)	170 (37.9)	1.00	1.00
Recovery with worsening health	72 (58.1)	52 (41.9)	1.18 (0.78 to 1.78)	1.05 (0.69 to 1.61)
PCS with improvement	178 (56.3)	138 (43.7)	1.26 (0.93 to 1.70)	1.10 (0.81 to 1.50)
Persistent PCS	342 (52.2)	313 (47.8)	1.56 (1.21 to 2.01)	1.21 (0.93 to 1.57)

[1] Adjusted for sex-age class combinations and study centre

[2] additionally adjusted for university entrance qualification, BMI and smoking status

FEU: Fibrinogen Equivalent Units
