# Supplementary material for: Persistent symptoms and clinical findings in adults with post-acute sequelae of COVID-19/post-COVID-19 syndrome in the second year after acute infection: A population-based, nested case-control study
Source: PLoS Med. 2025 Jan 23;22(1):e1004511. doi: 10.1371/journal.pmed.1004511 (PMC12005676; doi:10.1371/journal.pmed.1004511)
Supplement: S7 Table — (PDF) [file pmed.1004511.s012.pdf]

S7 Table. Phase 2 case-control status by EBV and CMV antibody pattern.

	EBV antibodies					CMV antibodies				
	N (%)			Reactivated vs. previously infected		N (%)			Positive 23-199 vs. ≥200 RE/ml	
	Negative[1]	Previously infected[2]	Re-activated[3]	OR (95%-CI) [4]	OR_{Adj} (95%-CI) [5]	Negative <22 RE/ml	Positive 23-199 RE/ml	Positive ≥200 RE/ml	OR (95%-CI)[4]	OR_{Adj} (95%-CI)[5]
Continued recovery	22 (4.9)	372 (83.2)	53 (11.9)	1.00	1.00	271 (60.6)	146 (32.7)	30 (6.7)	1.00	1.00
Recovery with worsening health	5 (4.0)	106 (86.2)	12 (9.8)	0.77 (0.40 to 1.50)	0.77 (0.40 to 1.51)	78 (63.4)	35 (28.5)	10 (8.1)	1.34 (0.59 to 3.06)	1.31 (0.57 to 2.99)
PCS with improvement	15 (4.9)	258 (83.5)	36 (11.7)	1.00 (0.63 to 1.58)	1.00 (0.63 to 1.58)	189 (61.0)	99 (31.9)	22 (7.1)	1.13 (0.61 to 2.10)	1.11 (0.60 to 2.05)
Persistent PCS	24 (3.7)	538 (82.4)	93 (14.2)	1.20 (0.83 to 1.72)	1.20 (0.83 to 1.74)	380 (57.9)	218 (33.2)	58 (8.8)	1.32 (0.80 to 2.16)	1.27 (0.77 to 1.10)

[1] Negative: VCA <22 RE/ml and EBNA <22 RE/ml and EA-D <22 RE/ml

[2] Previously infected: (VCA ≥22 RE/ml or EBNA ≥22 RE/ml) and EA-D <22 RE/ml

[3] Reactivated: (VCA ≥22 RE/ml or EBNA ≥22 RE/ml) and EA-D ≥22 RE/ml

[4] Adjusted for sex-age class combinations and study centre

[5] additionally adjusted for university entrance qualification

EBV: Epstein-Barr virus

VCA: Viral Capsid Antigen

EA-D: Early Antigen-Diffuse

EBNA: Epstein-Barr Nuclear Antigen

CMV: Cytomegalovirus
