# Supplementary figures and images for: Persistent symptoms and clinical findings in adults with post-acute sequelae of COVID-19/post-COVID-19 syndrome in the second year after acute infection: A population-based, nested case-control study
Source: PLoS Med. 2025 Jan 23;22(1):e1004511. doi: 10.1371/journal.pmed.1004511 (PMC12005676; doi:10.1371/journal.pmed.1004511)

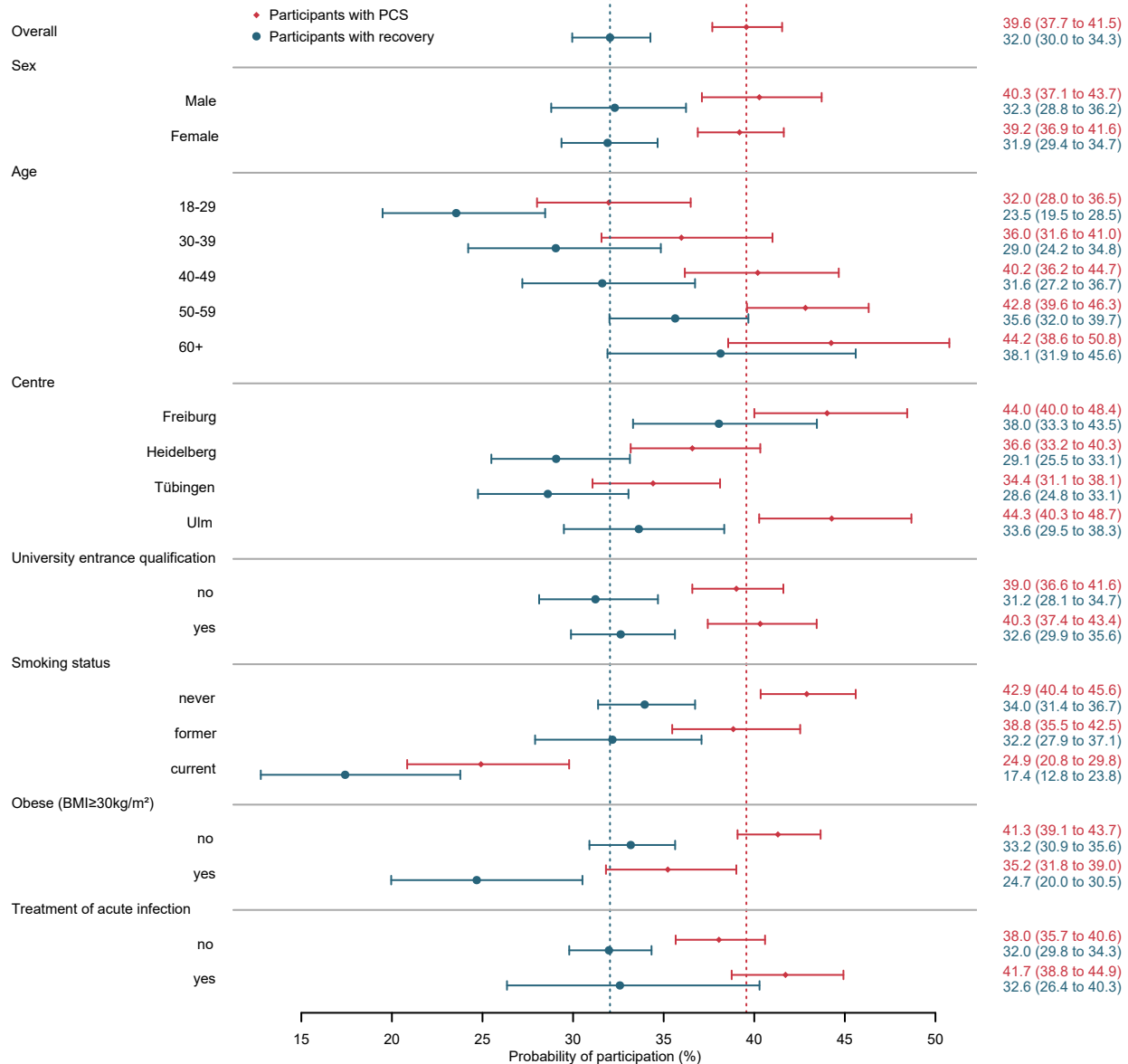

**S1 Fig.** Probability of participation (95% CI) by selected phase 1 characteristics.

Supplement: S1 Fig — (PDF) [file pmed.1004511.s013.pdf]
