# Supplementary material for: Persistent symptoms and clinical findings in adults with post-acute sequelae of COVID-19/post-COVID-19 syndrome in the second year after acute infection: A population-based, nested case-control study
Source: PLoS Med. 2025 Jan 23;22(1):e1004511. doi: 10.1371/journal.pmed.1004511 (PMC12005676; doi:10.1371/journal.pmed.1004511)
Supplement: S2 Fig — (PDF) [file pmed.1004511.s014.pdf]

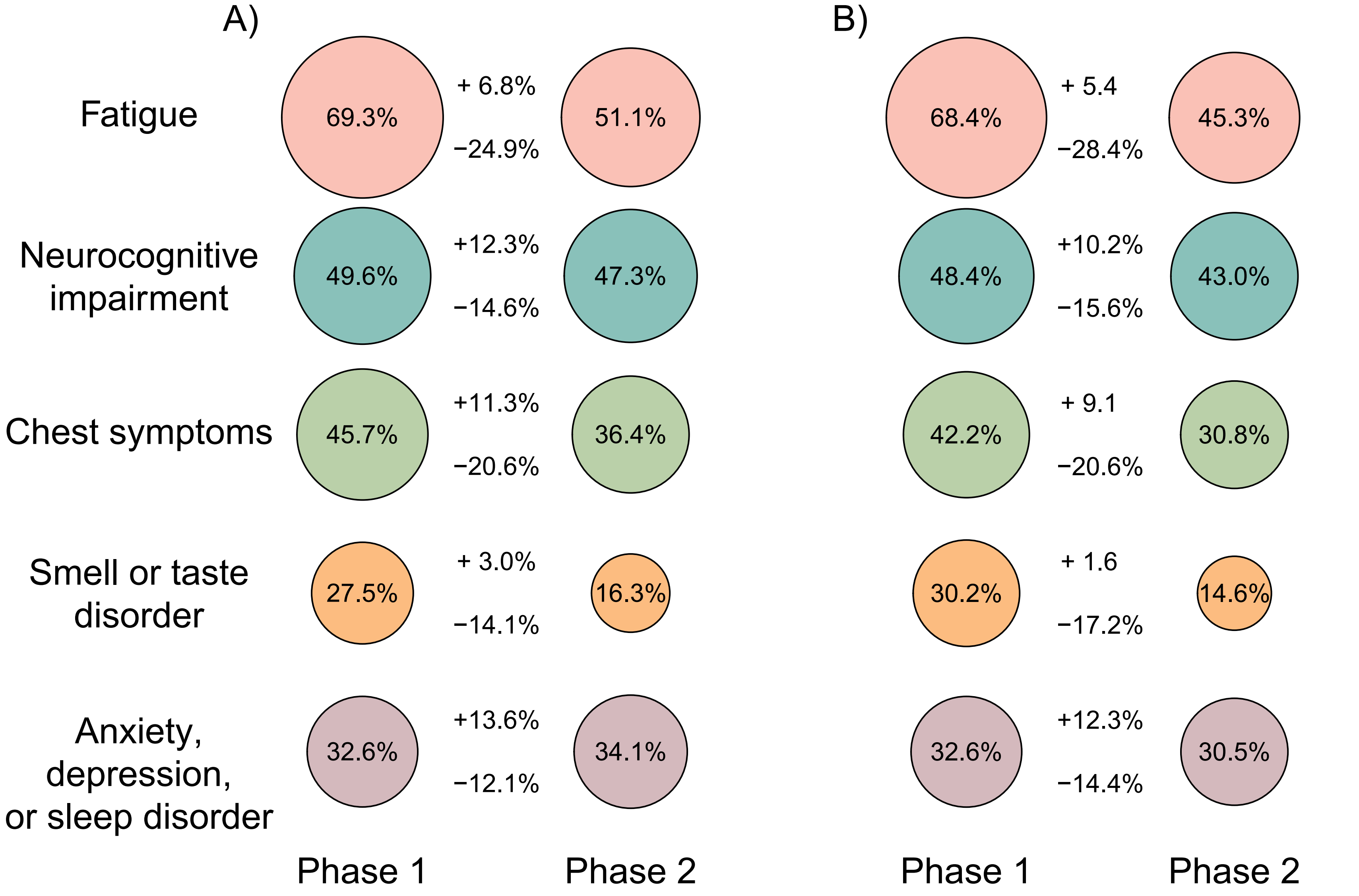

S2 Fig. Changes in the prevalence of the five main symptom clusters (based on self-reported new symptoms of moderate to strong severity after acute infection) in phase 1 participants with PCS participating in phase 2. A) all participants. B) excluding participants with health conditions already present before index infection (cardiovascular diseases, respiratory diseases, mental disorders, neurologic or sensory disorders, cancer, metabolic diseases, n=599) and cases with an alternative explanation of persisting symptoms (n=41).
