# Supplementary material for: Persistent symptoms and clinical findings in adults with post-acute sequelae of COVID-19/post-COVID-19 syndrome in the second year after acute infection: A population-based, nested case-control study
Source: PLoS Med. 2025 Jan 23;22(1):e1004511. doi: 10.1371/journal.pmed.1004511 (PMC12005676; doi:10.1371/journal.pmed.1004511)
Supplement: S3 Fig — (PDF) [file pmed.1004511.s015.pdf]

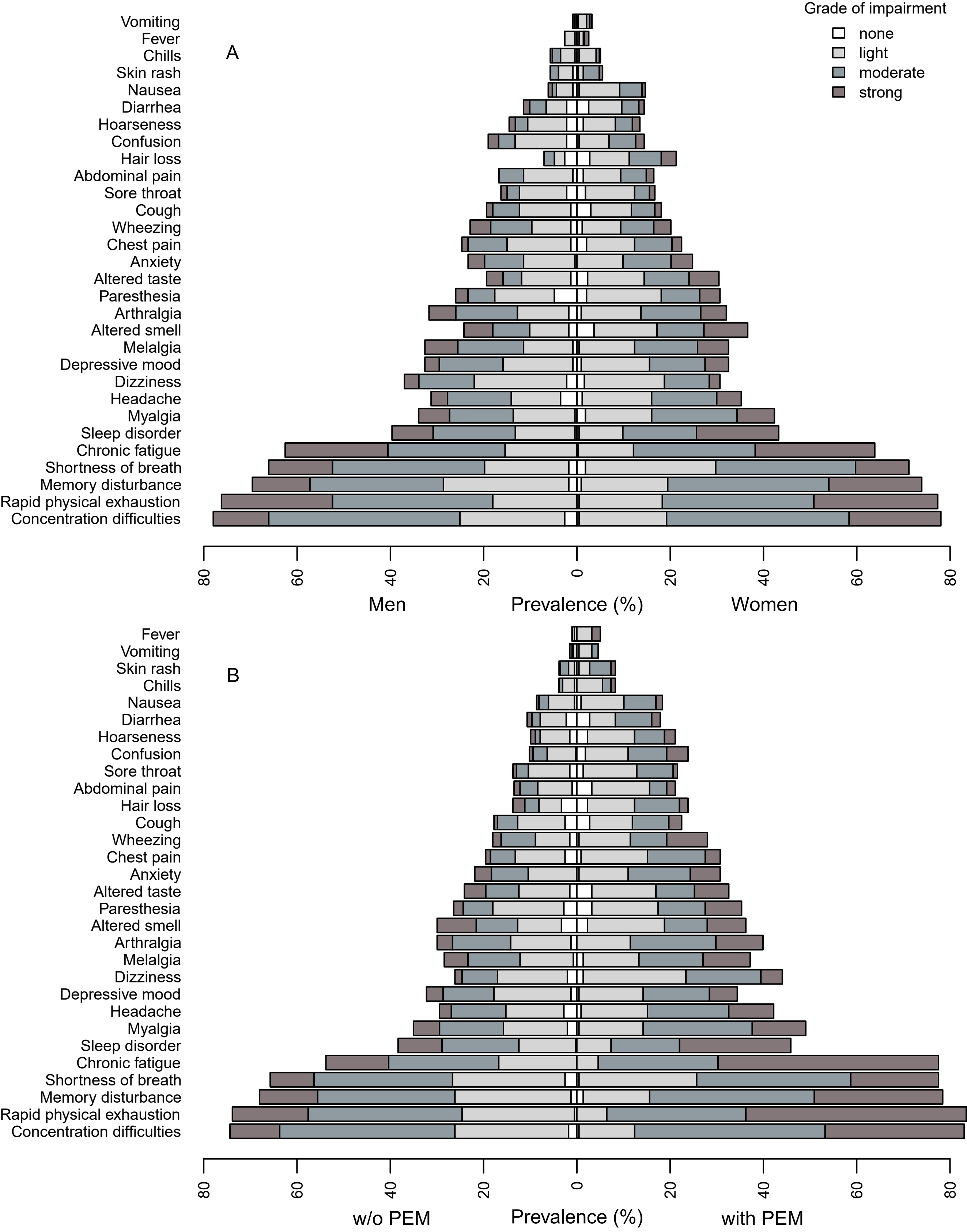

S3 Fig. Individual symptoms of different grades among (A) male and female participants with persistent PCS, (B) among participants with persistent PCS with or without post-exertional malaise (PEM), lasting >14 hours.
