# Supplementary material for: Persistent symptoms and clinical findings in adults with post-acute sequelae of COVID-19/post-COVID-19 syndrome in the second year after acute infection: A population-based, nested case-control study
Source: PLoS Med. 2025 Jan 23;22(1):e1004511. doi: 10.1371/journal.pmed.1004511 (PMC12005676; doi:10.1371/journal.pmed.1004511)
Supplement: S5 Fig — (PDF) [file pmed.1004511.s017.pdf]

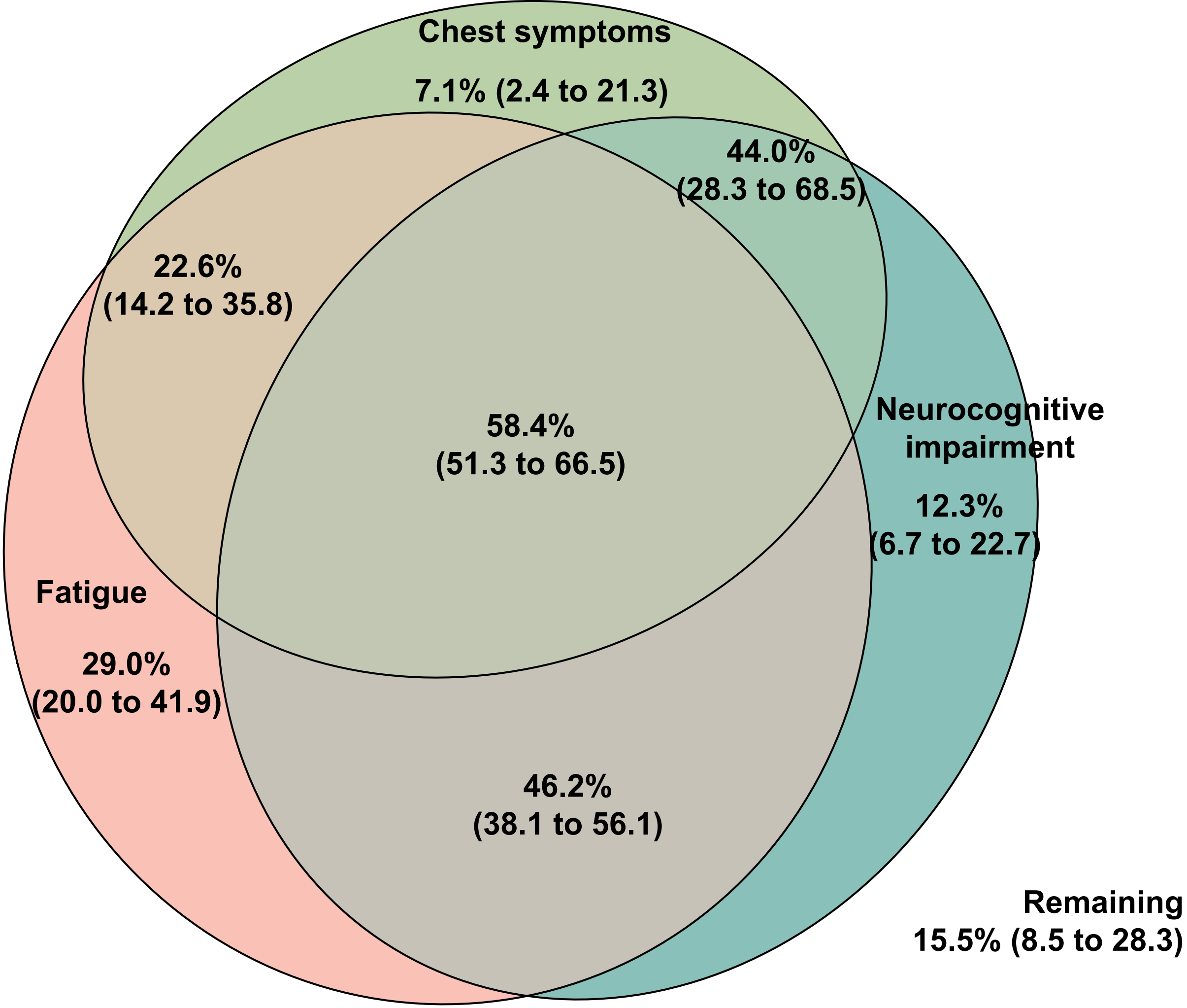

S5 Fig. Euler graphs showing the descriptive prevalence (with 95%CI) of post exertional malaise (PEM, lasting >14 hours) in the various overlaps of the three main symptom clusters from phase 2 based on symptoms of grade moderate to strong in participants with persistent PCS only.
