# Supplementary material for: Persistent symptoms and clinical findings in adults with post-acute sequelae of COVID-19/post-COVID-19 syndrome in the second year after acute infection: A population-based, nested case-control study
Source: PLoS Med. 2025 Jan 23;22(1):e1004511. doi: 10.1371/journal.pmed.1004511 (PMC12005676; doi:10.1371/journal.pmed.1004511)
Supplement: S6 Fig — (PDF) [file pmed.1004511.s018.pdf]

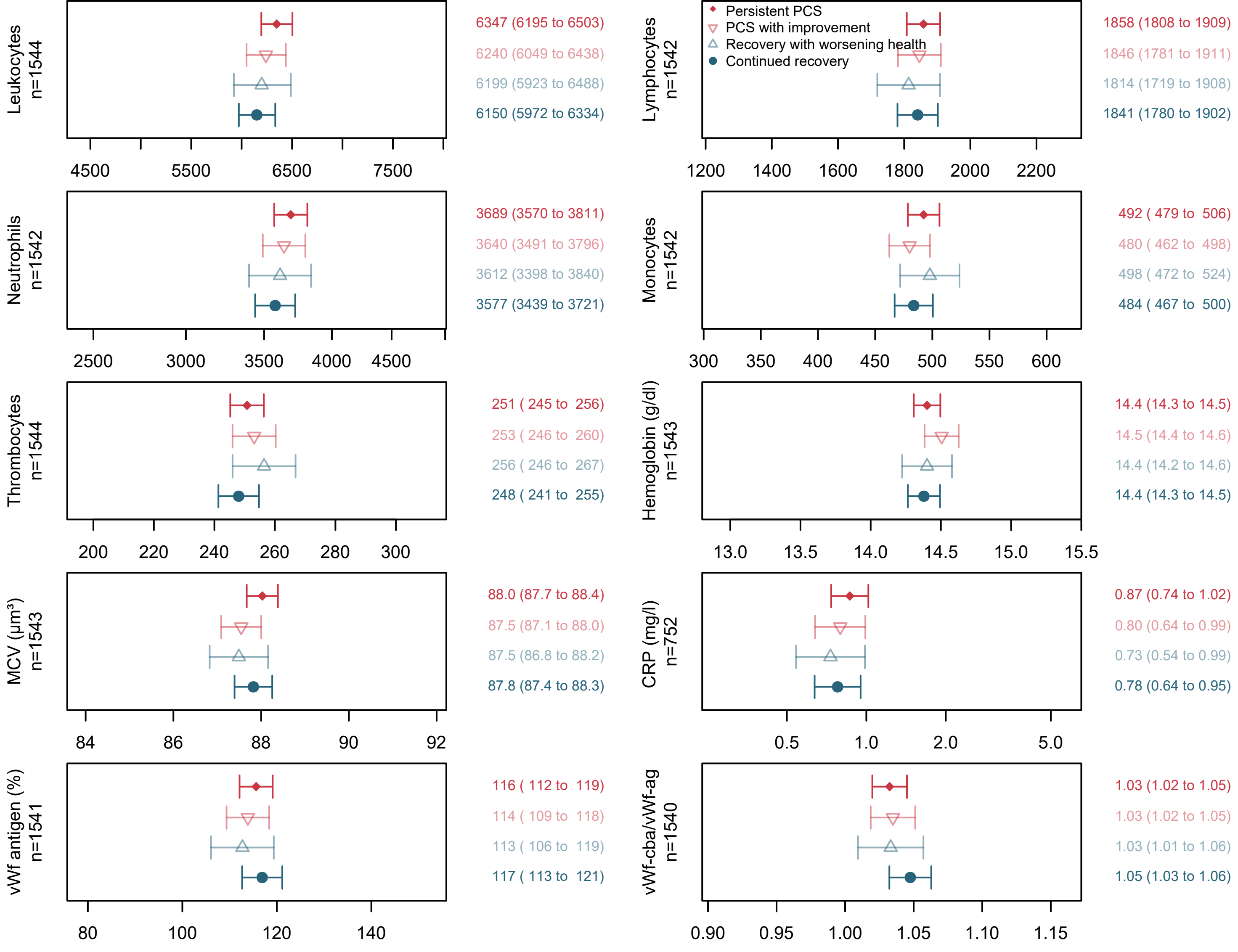

S6 Fig. Means (geometric mean for CRP) of blood cell counts (with 95%-CI) by case-control status at clinical examination in phase 2. Adjusted for sex-age class combinations, study centre, university entrance qualification. For comparability the x-axis is scaled from mean -1 SD to mean +1 SD for all panels.
