# Supplementary material for: Persistent symptoms and clinical findings in adults with post-acute sequelae of COVID-19/post-COVID-19 syndrome in the second year after acute infection: A population-based, nested case-control study
Source: PLoS Med. 2025 Jan 23;22(1):e1004511. doi: 10.1371/journal.pmed.1004511 (PMC12005676; doi:10.1371/journal.pmed.1004511)
Supplement: S7 Fig — (PDF) [file pmed.1004511.s019.pdf]

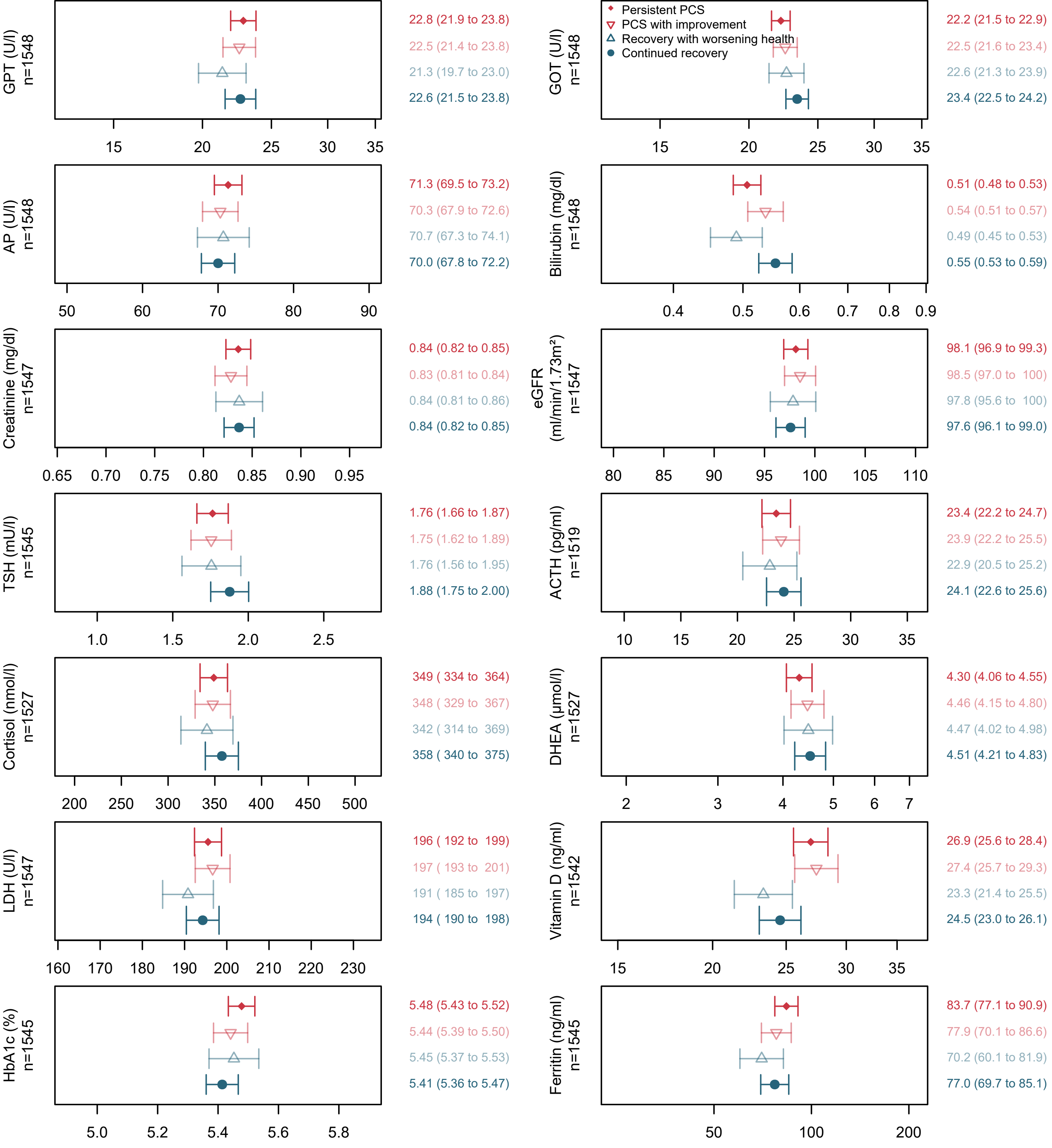

S7 Fig. Mean (geometric mean for GPT, GOT, bilirubin, DHEA-S, vitamin D, and ferritin) of selected laboratory measurements (with 95%-CI) by case-control status at clinical examina on in phase 2. Adjusted for sex-age class combinations, study centre, university entrance qualification, BMI and smoking status. ACTH, cortisol and DHEA-S were additionally adjusted for time of sampling; and vitamin D for intake of vitamin D supplements. For comparability the x-axis is scaled from mean -1 SD to mean +1 SD for all panels.
