# Supplementary material for: Persistent symptoms and clinical findings in adults with post-acute sequelae of COVID-19/post-COVID-19 syndrome in the second year after acute infection: A population-based, nested case-control study
Source: PLoS Med. 2025 Jan 23;22(1):e1004511. doi: 10.1371/journal.pmed.1004511 (PMC12005676; doi:10.1371/journal.pmed.1004511)
Supplement: S8 Fig — (PDF) [file pmed.1004511.s020.pdf]

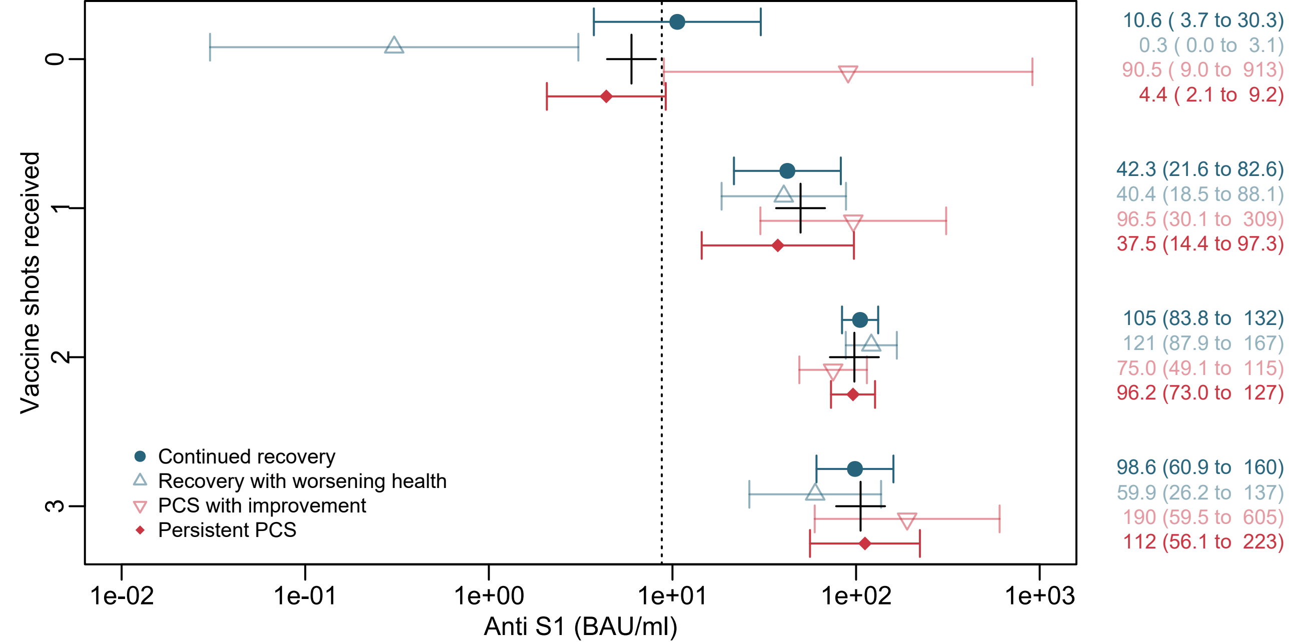

S8 Fig. Geometric mean of anti S1 titer (BAU/ml) by number of received vaccine shots and case-control status at clinical examination in phase 1, adjusted for sex-age class combinations, study centre, and university entrance qualification. Quantitative data were determined by dilution of samples for one centre only (N=398). Values left of the dotted line (below 8.75 BAU/ml) are considered negative; crosses represent the geometric mean per number of received vaccine shots independent of case-control status.
