# Supplementary material for: Persistent symptoms and clinical findings in adults with post-acute sequelae of COVID-19/post-COVID-19 syndrome in the second year after acute infection: A population-based, nested case-control study
Source: PLoS Med. 2025 Jan 23;22(1):e1004511. doi: 10.1371/journal.pmed.1004511 (PMC12005676; doi:10.1371/journal.pmed.1004511)
Supplement: S9 Fig — (PDF) [file pmed.1004511.s021.pdf]

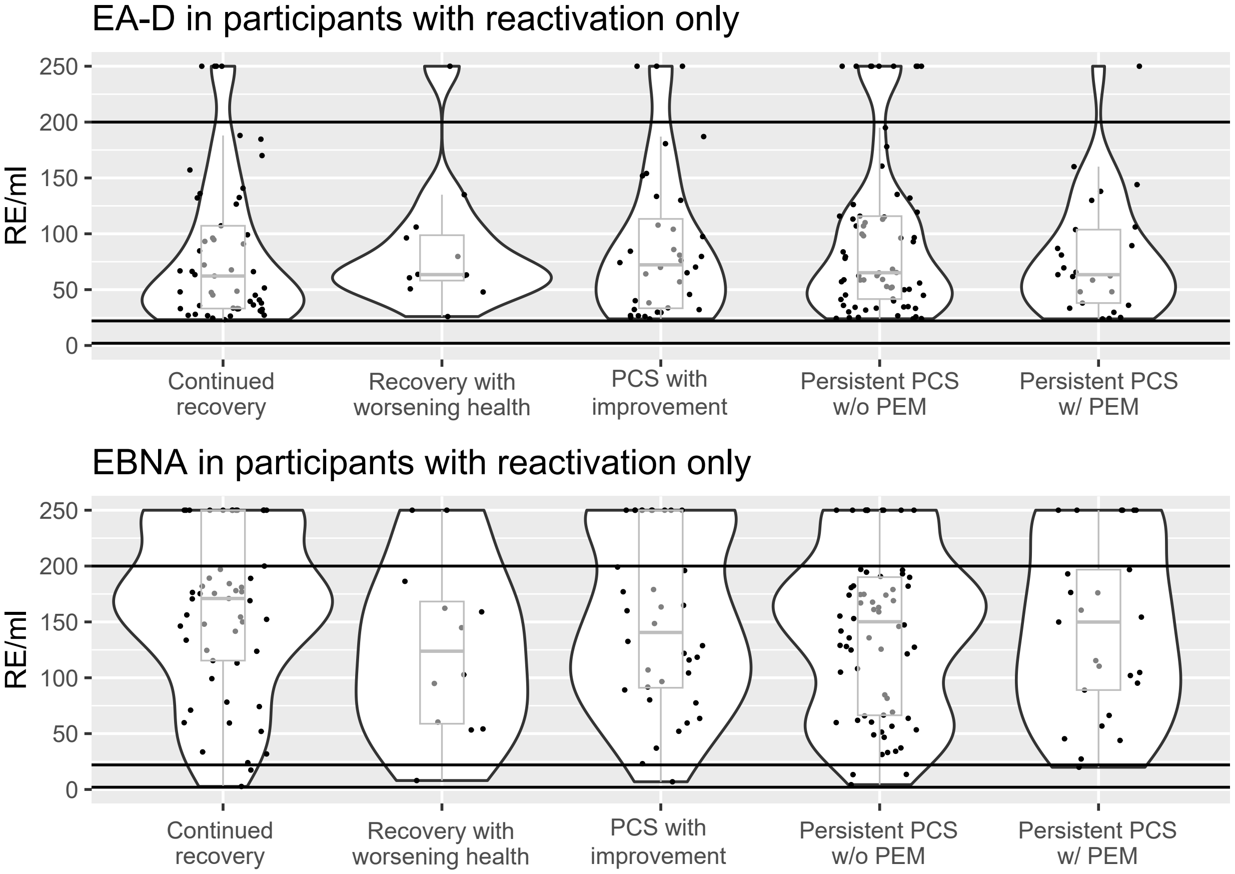

S9 Fig. EA-D and EBNA IgG antibody levels in participants with evidence for EBV reactivation.
