# Supplementary material for: Persistent symptoms and clinical findings in adults with post-acute sequelae of COVID-19/post-COVID-19 syndrome in the second year after acute infection: A population-based, nested case-control study
Source: PLoS Med. 2025 Jan 23;22(1):e1004511. doi: 10.1371/journal.pmed.1004511 (PMC12005676; doi:10.1371/journal.pmed.1004511)
Supplement: S10 Fig — (PDF) [file pmed.1004511.s022.pdf]

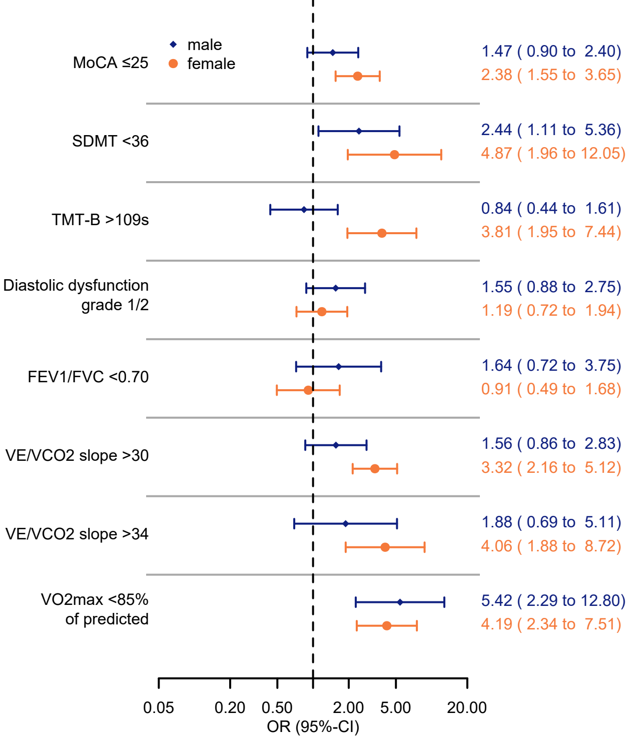

S10 Fig. Sex specific association of case-control status (Persistent PCS vs. Continued recovery) with abnormal neurocognitive and cardiopulmonary test results.
