# Supplementary material for: Persistent symptoms and clinical findings in adults with post-acute sequelae of COVID-19/post-COVID-19 syndrome in the second year after acute infection: A population-based, nested case-control study
Source: PLoS Med. 2025 Jan 23;22(1):e1004511. doi: 10.1371/journal.pmed.1004511 (PMC12005676; doi:10.1371/journal.pmed.1004511)
Supplement: S1 Sensitivity Analyses — (PDF) [file pmed.1004511.s023.pdf]

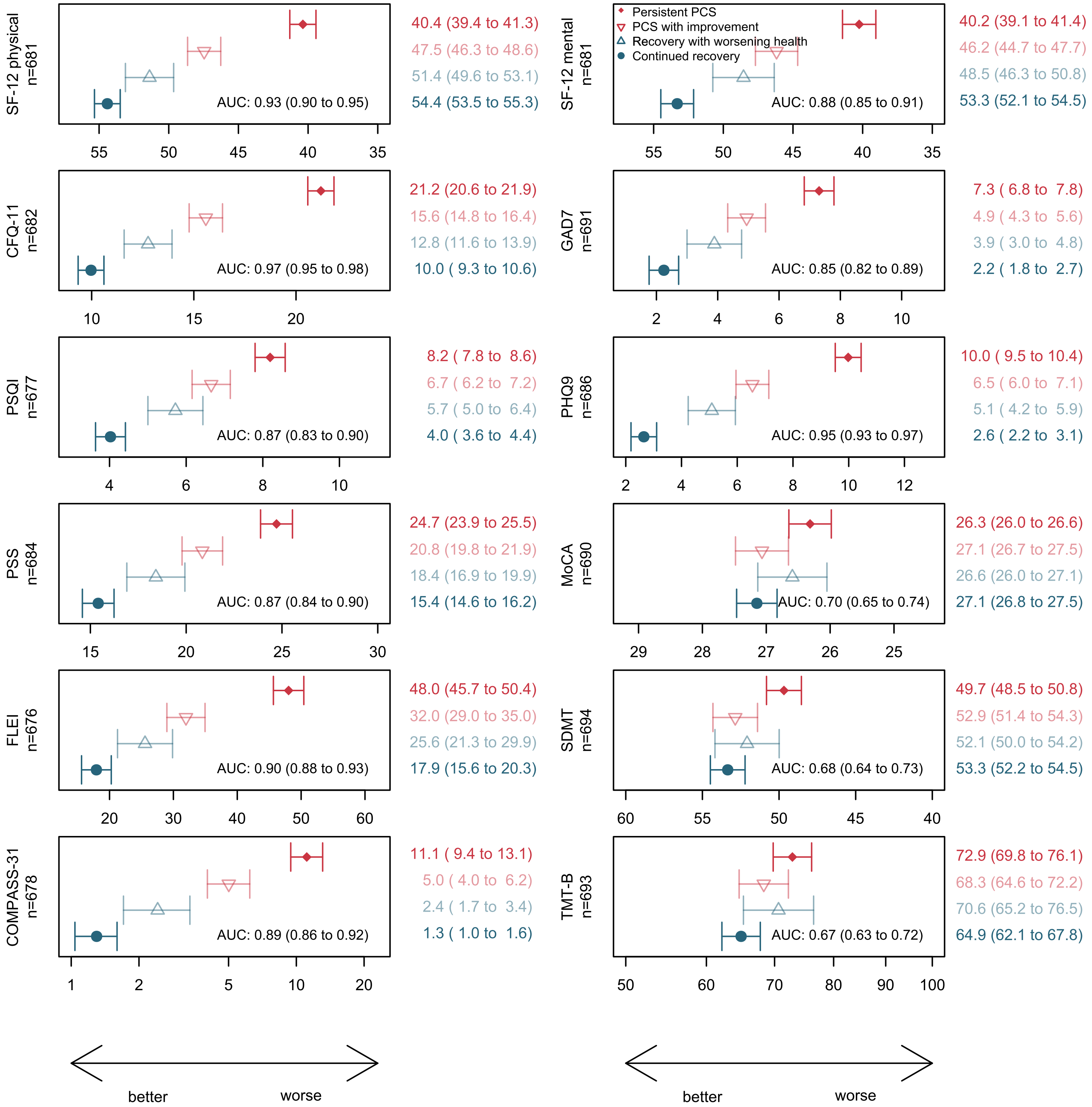

Figure A. Sensitivity analysis 1, excluding participants with health conditions already present before index infection (cardiovascular diseases, respiratory diseases, mental disorders, neurologic or sensory disorders, cancer, metabolic diseases, n=599) and participants with a possible alternative medical explanation of persisting symptoms (n=41). Shown are means (geometric mean for COMPASS-31 and TMT-B) of self-reported health outcomes and neurocognitive tests (with 95%-CI) by case-control status at clinical examination in phase 2, adjusted for sex-age class combinations, study centre, and university entrance qualification. The reported area under the curve (AUC) for participants with persistent PCS vs. participants with continued recovery by the respective instrument is adjusted for sex-age class combinations and university entrance qualification. For comparability the x-axis is scaled from mean -1 SD to mean +1 SD for all panels. MoCA: Montreal cognitive assessment scale (points); SDMT: Symbol Digit Modalities Test (number of correct symbols); TMT-B: Trail making test B (time in seconds).

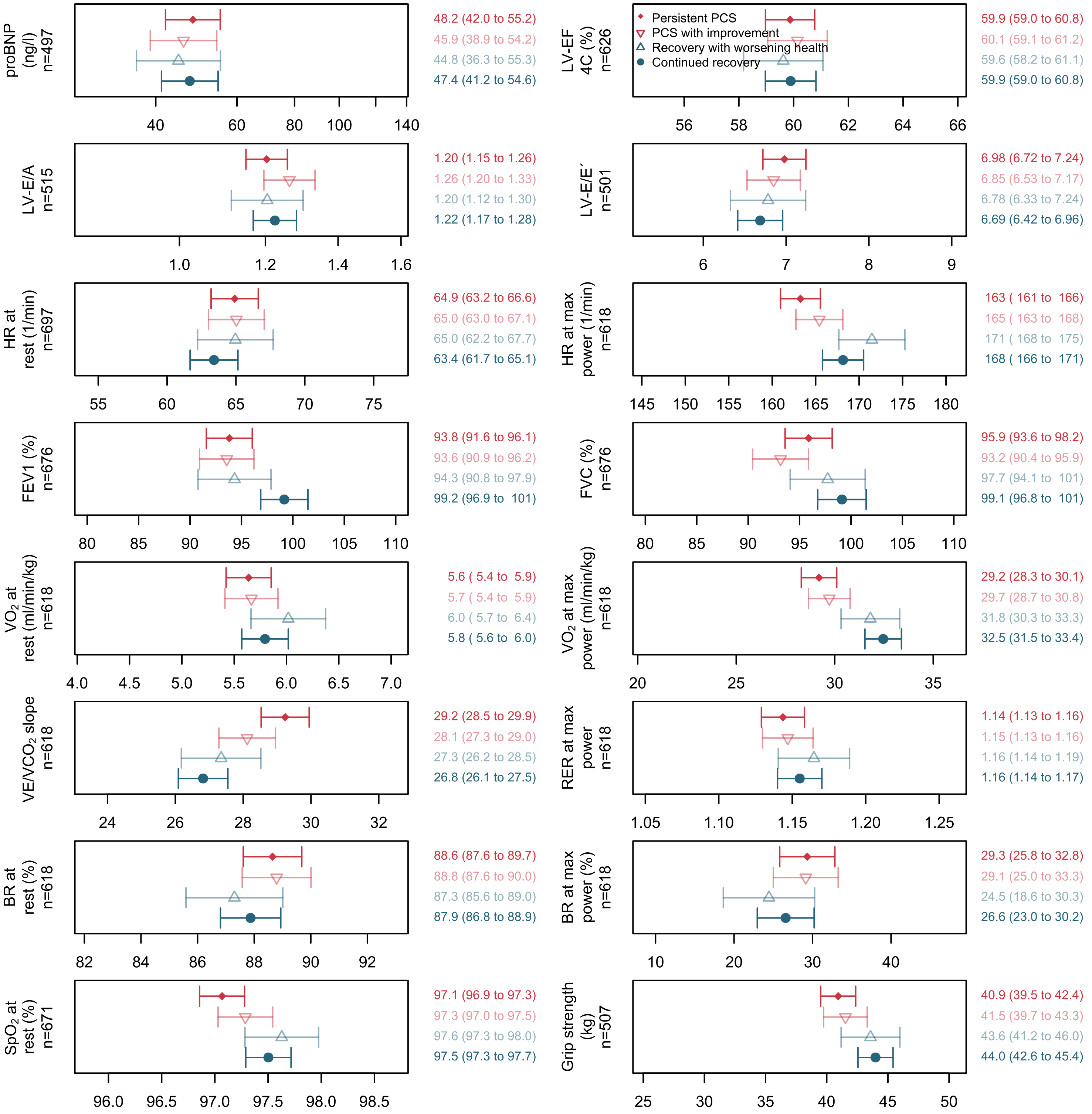

Figure B. Sensitivity analysis 1, excluding participants with health conditions already present before index infection (cardiovascular diseases, respiratory diseases, mental disorders, neurologic or sensory disorders, cancer, metabolic diseases, n=599) and participants with an alternative explanation of persisting symptoms (n=41). Shown are cardiopulmonary function indicators and grip strength (means with 95%-CI) by case-control status at clinical examination in phase 2. Adjusted for sex-age class combinations, study centre, university entrance qualification, BMI, smoking status and use of beta blocking agents. For comparability the x-axis is scaled from mean -1 SD to mean +1 SD for all panels.

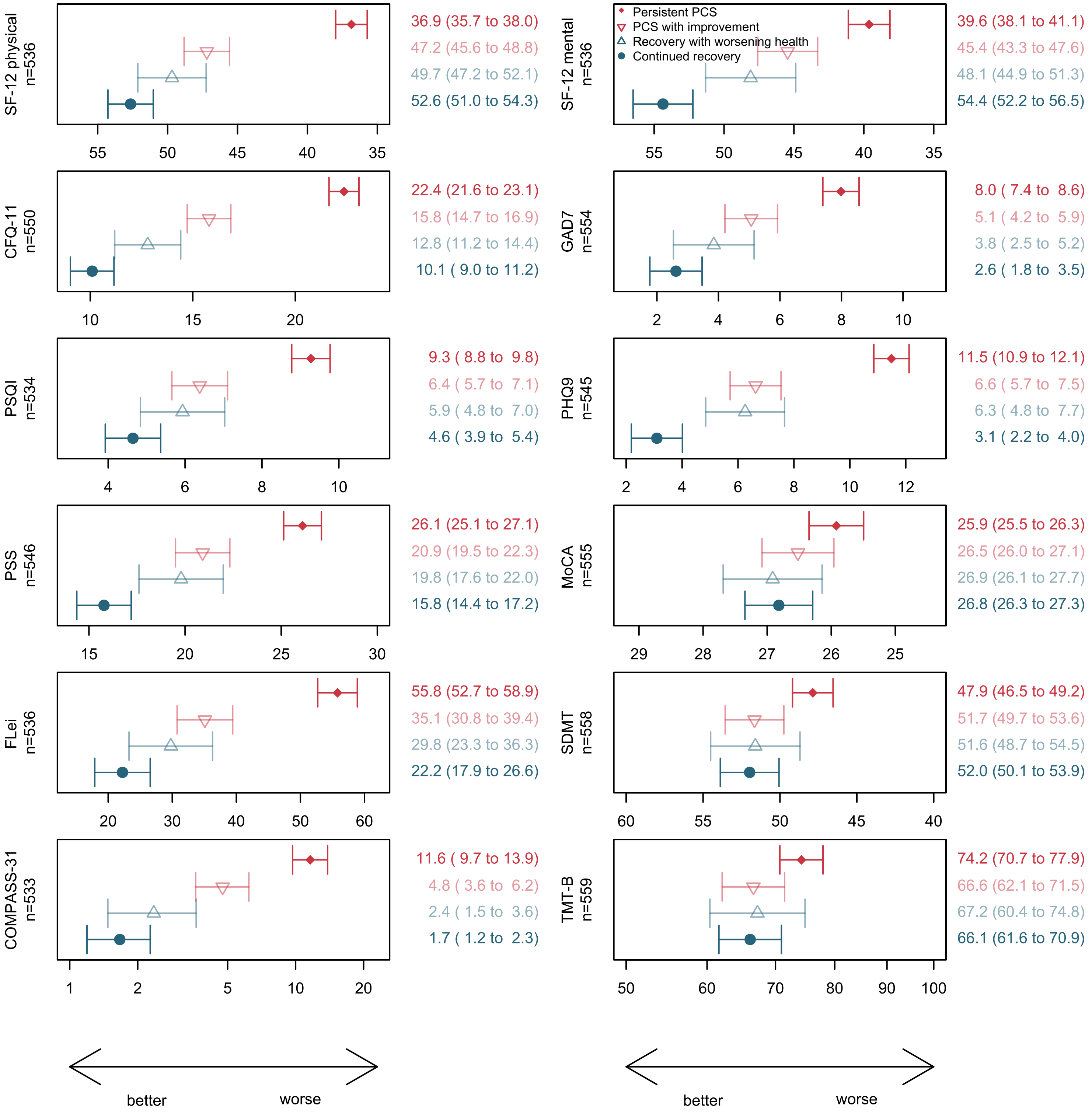

Figure C. Sensitivity analysis 2, showing results for study participants with a BMI ≥27.5 kg/m². Shown are means (geometric mean for COMPASS-31 and TMT-B) of self-reported health outcomes and neurocognitive tests (with 95%-CI) by case-control status at clinical examination in phase 2, adjusted for sex-age class combinations, study centre, and university entrance qualification. For comparability the x-axis is scaled from mean -1 SD to mean +1 SD for all panels. MoCA: Montreal cognitive assessment scale (points); SDMT: Symbol Digit Modalities Test (number of correct symbols); TMT-B: Trail making test B (time in seconds).

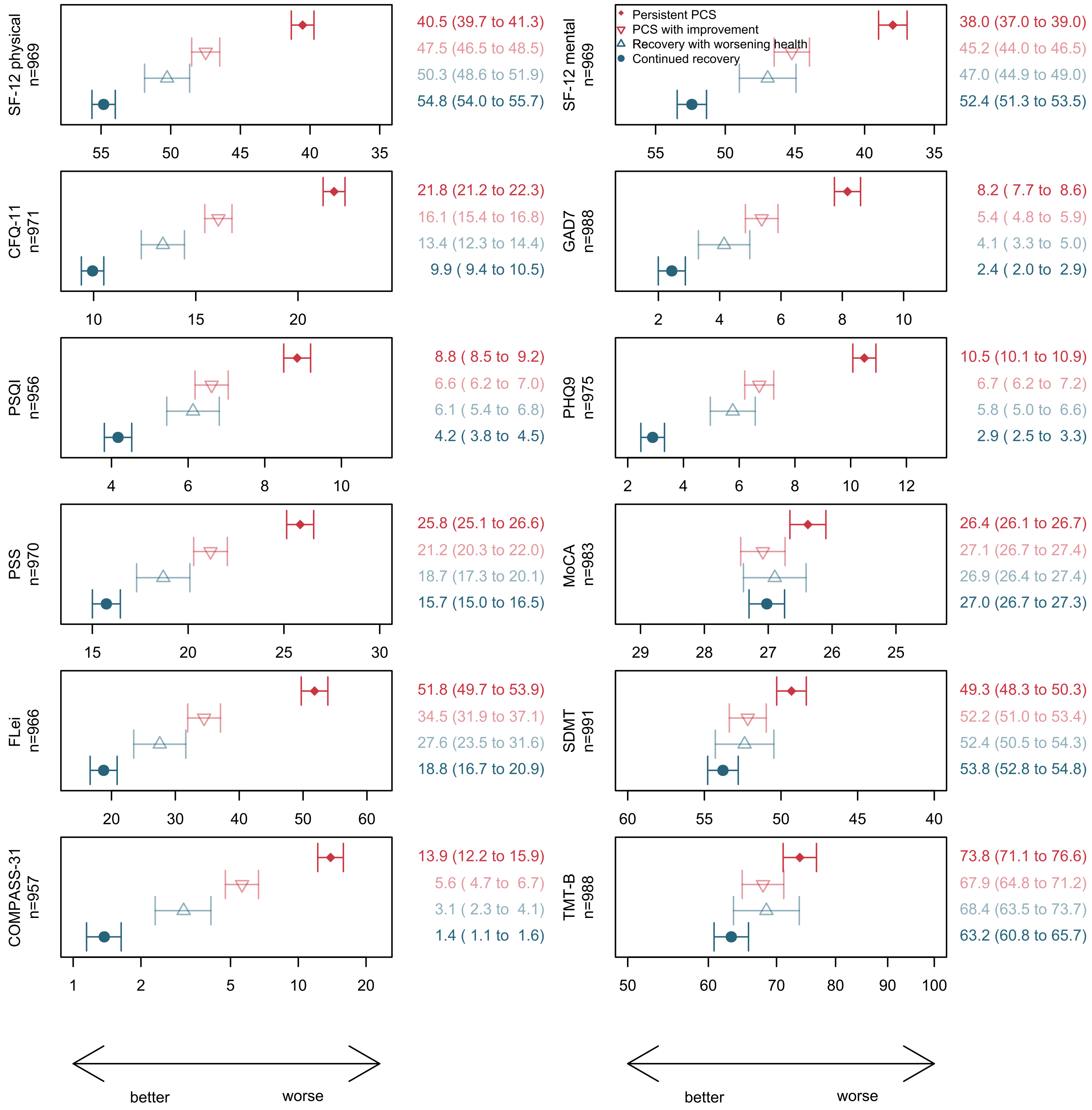

Figure D. Sensitivity analysis 2, results for study participants with a BMI <27.5 kg/m². Shown are means (geometric mean for COMPASS-31 and TMT-B) of self-reported health outcomes and neurocognitive tests (with 95%-CI) by case-control status at clinical examination in phase 2, adjusted for sex-age class combinations, study centre, and university entrance qualification. For comparability the x-axis is scaled from mean -1 SD to mean +1 SD for all panels. MoCA: Montreal cognitive assessment scale (points); SDMT: Symbol Digit Modalities Test (number of correct symbols); TMT-B: Trail making test B (time in seconds).

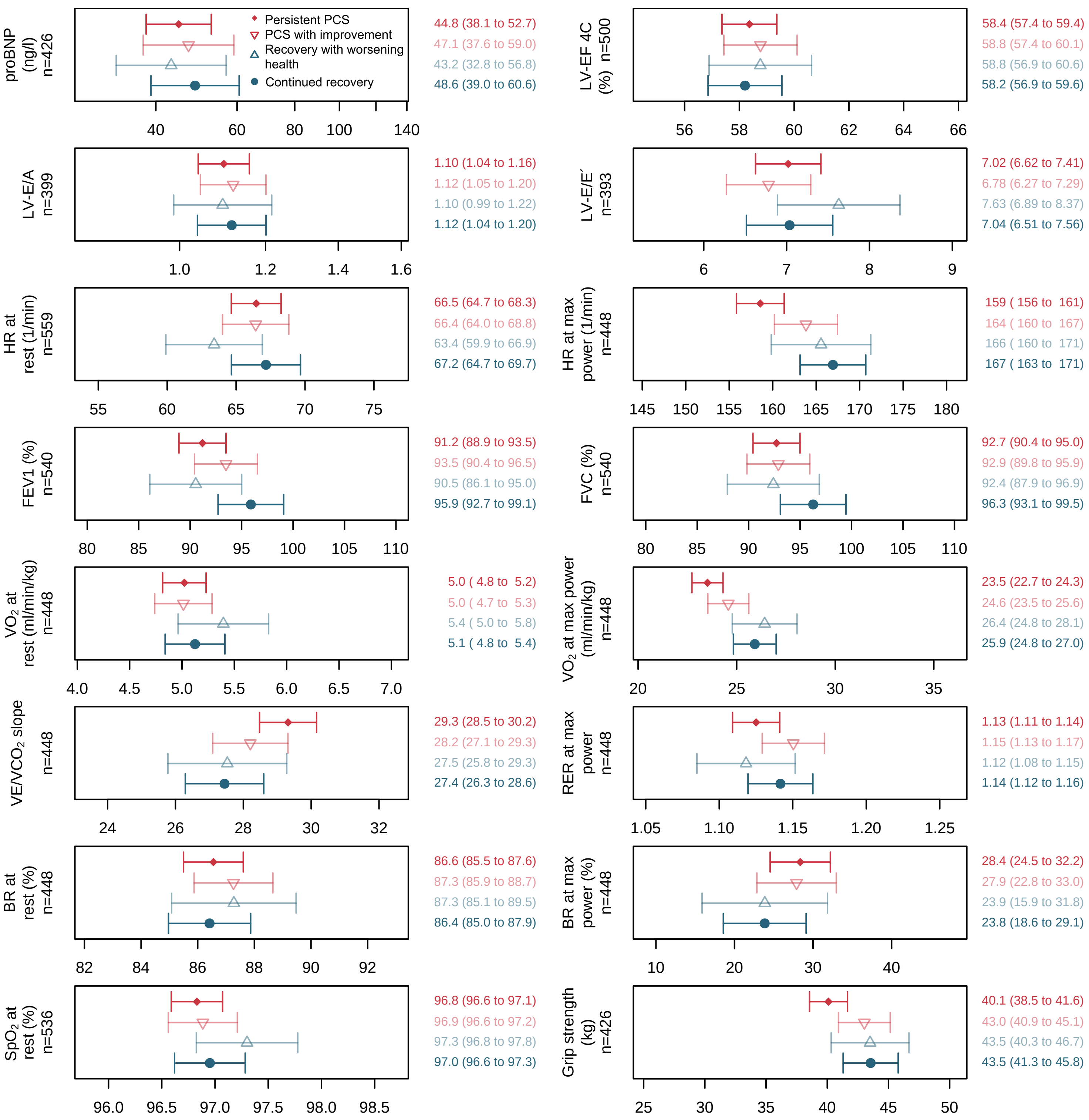

Figure E. Sensitivity analysis 2, results for study participants with a BMI ≥27.5 kg/m². Shown are cardiopulmonary function indicators and grip strength (means with 95%-CI) by case-control status at clinical examination in phase 2. Adjusted for sex-age class combinations, study centre, university entrance qualification, smoking status and use of beta blocking agents. For comparability the x-axis is scaled from mean -1 SD to mean +1 SD for all panels.

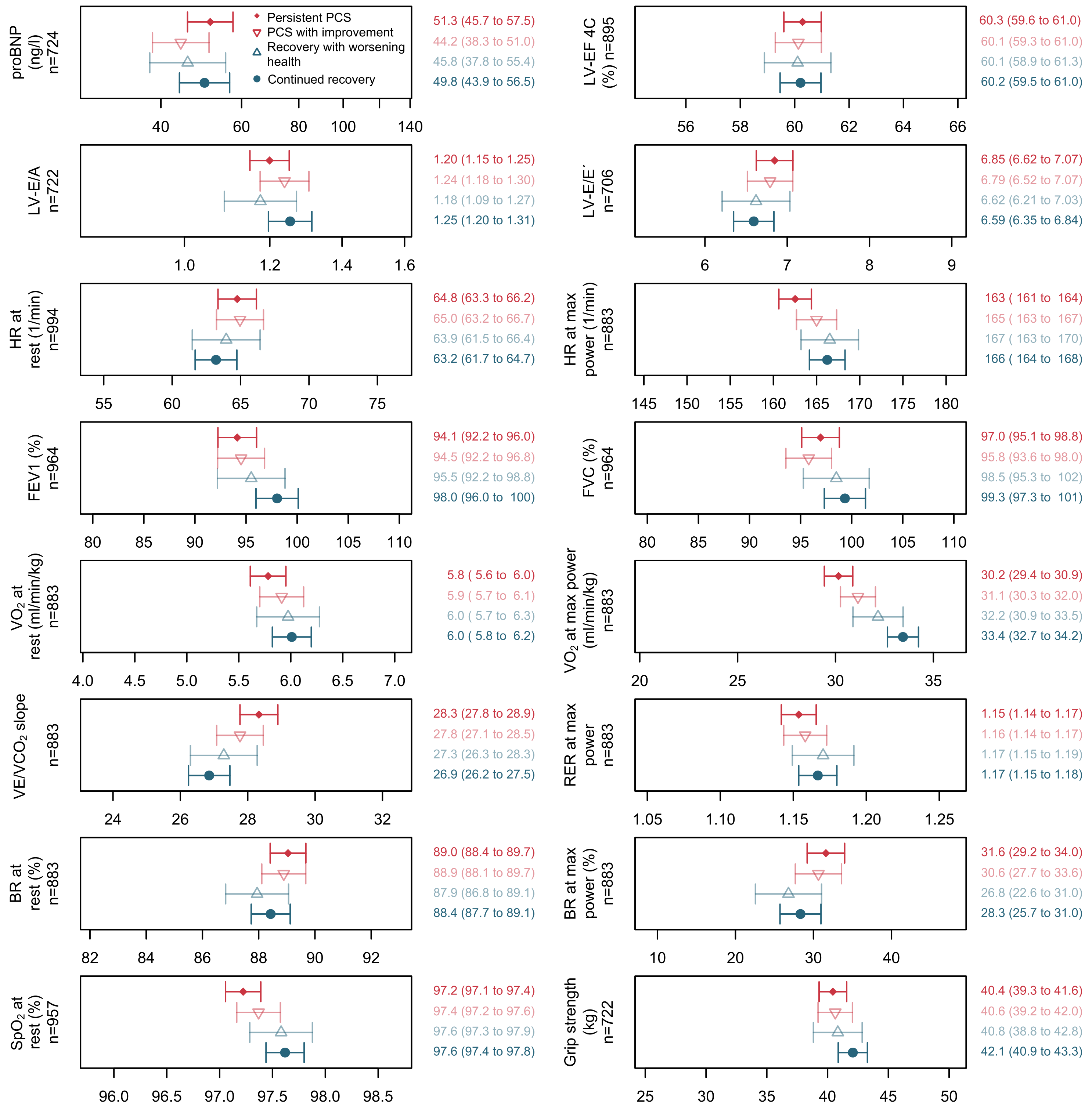

Figure F. Sensitivity analysis 2, results for study participants with a BMI <27.5 kg/m². Shown are cardiopulmonary function indicators and grip strength (means with 95%-CI) by case-control status at clinical examination in phase 2. Adjusted for sex-age class combinations, study centre, university entrance qualification, smoking status and use of beta blocking agents. For comparability the x-axis is scaled from mean -1 SD to mean +1 SD for all panels.

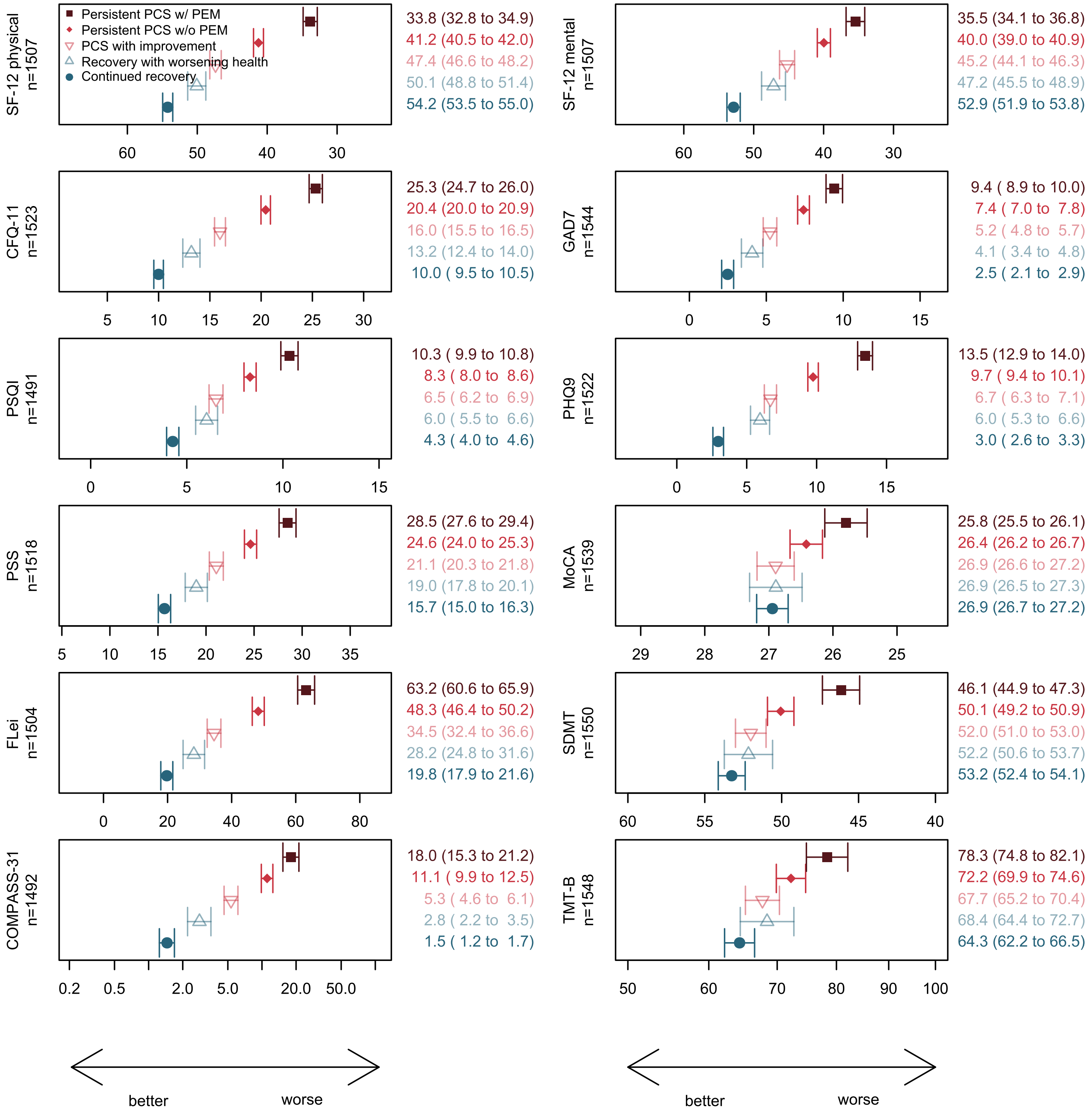

Figure G. Sensitivity analysis 3 with persistent PCS additionally stratified by presence of post-exertional malaise (PEM, lasting >14 hours). Shown are means (geometric mean for COMPASS-31 and TMT-B) of self-reported health outcomes and neurocognitive tests (with 95%-CI) at clinical examination in phase 2, adjusted for sex-age class combinations, study centre, and university entrance qualification. For comparability the x-axis is scaled from mean -2 SD to mean +2 SD for all panels. MoCA: Montreal cognitive assessment scale (points); SDMT: Symbol Digit Modalities Test (number of correct symbols); TMT-B: Trail making test B (time in seconds).

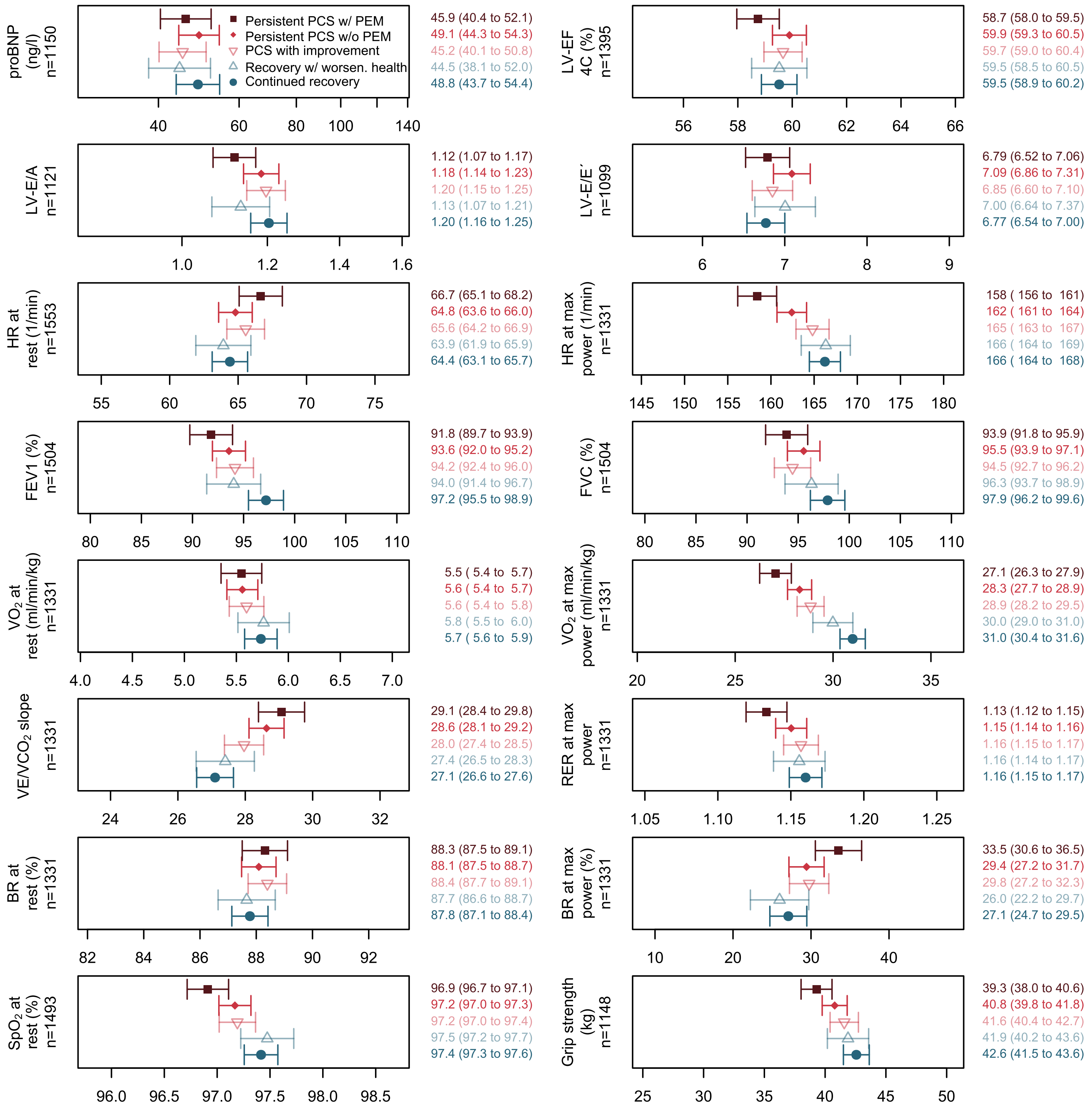

Figure H. Sensitivity analysis 3 with persistent PCS additionally stratified by presence of post-exertional malaise (PEM, lasting >14 hours). Shown are cardiopulmonary function indicators and handgrip strength (means with 95%-CI) at clinical examination in phase 2. Adjusted for sex-age class combinations, study centre, university entrance qualification, BMI, smoking status and use of beta blocking agents. For comparability the x-axis is scaled from mean -1 SD to mean +1 SD for all panels.

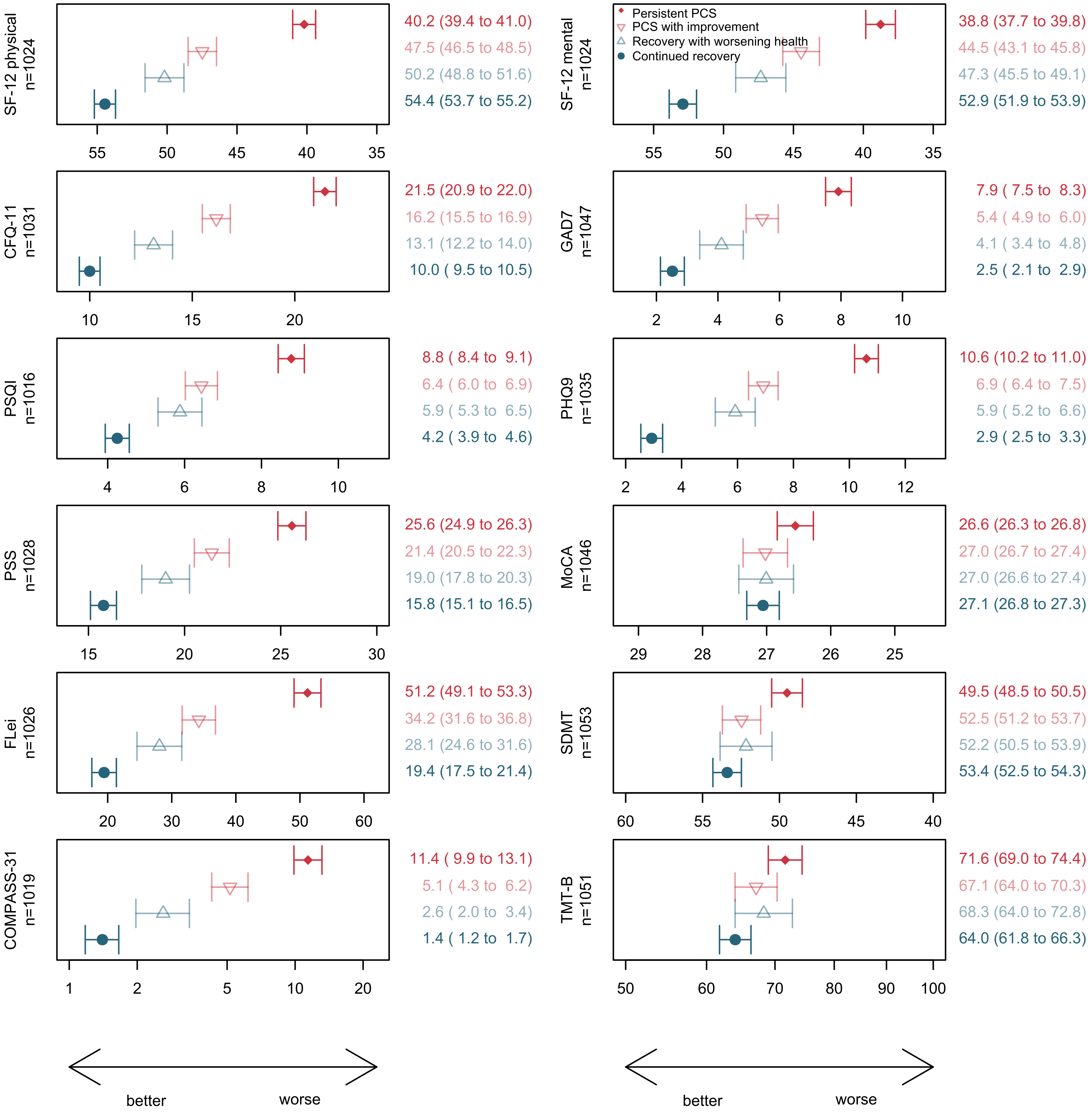

Figure I. Sensitivity analysis 4, in participants without medical care for their earlier acute (index) SARS-CoV-2 infection. Shown are means (geometric mean for COMPASS-31) of self-reported health outcomes (with 95%-CI) by case-control status at clinical examination in phase 2, adjusted for sex-age class combinations and university entrance qualification. For comparability the x-axis is scaled from mean -1 SD to mean +1 SD for all panels.

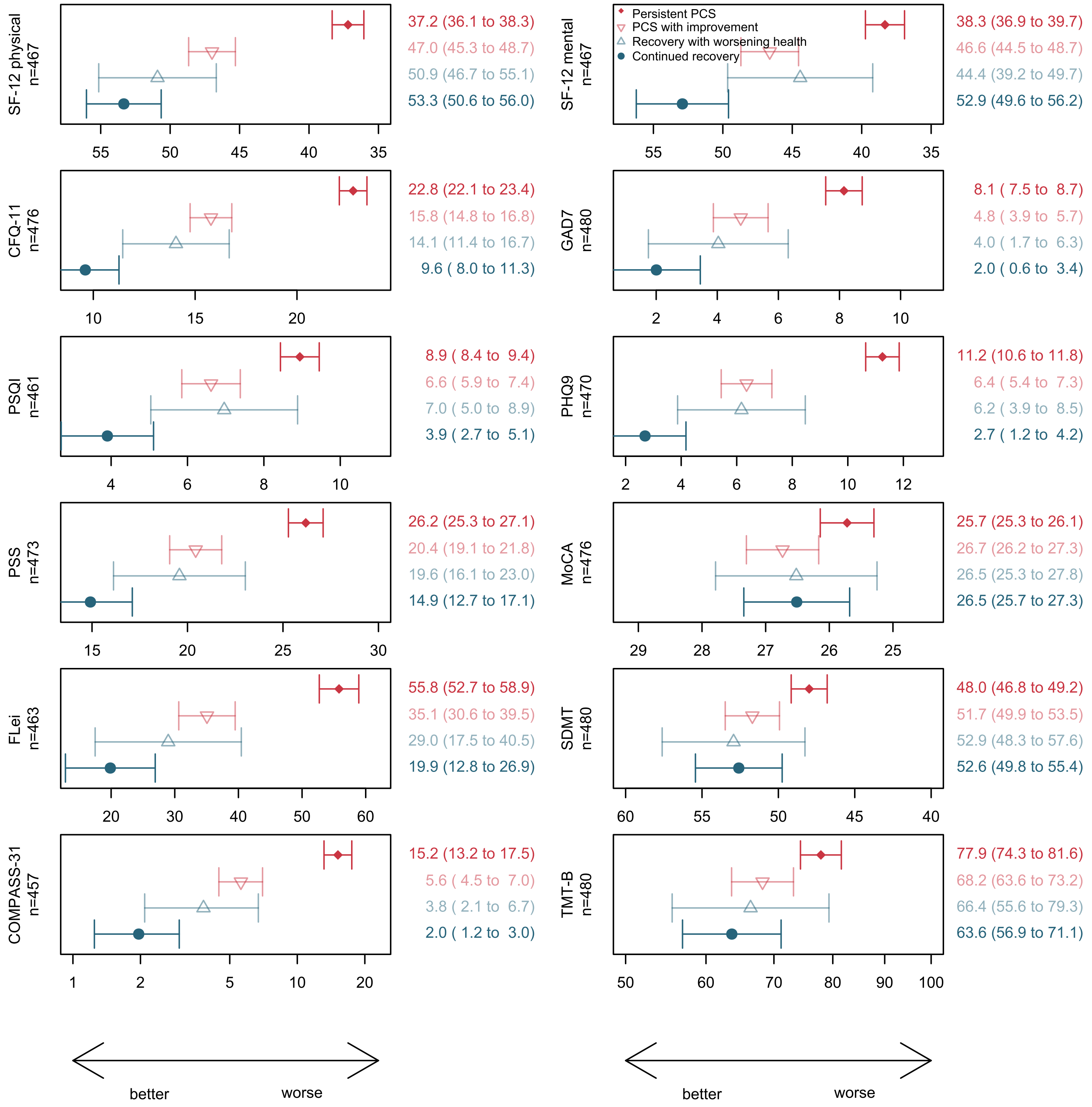

Figure J. Sensitivity analysis 4, in participants with medical care for their earlier acute (index) SARS-CoV-2 infection. Shown are means (geometric mean for COMPASS-31) of self-reported health outcomes (with 95%-CI) by case-control status at clinical examination in phase 2, adjusted for sex-age class combinations and university entrance qualification. For comparability the x-axis is scaled from mean -1 SD to mean +1 SD for all panels.
