# Supplementary material for: Persistent symptoms and clinical findings in adults with post-acute sequelae of COVID-19/post-COVID-19 syndrome in the second year after acute infection: A population-based, nested case-control study
Source: PLoS Med. 2025 Jan 23;22(1):e1004511. doi: 10.1371/journal.pmed.1004511 (PMC12005676; doi:10.1371/journal.pmed.1004511)
Supplement: Attachment — Submitted filename: EPILOC Ph2_REVISION_point-to-point response.docx [file pmed.1004511.s024.docx]

**(PMEDICINE-D-24-01834R1) to PLOS Medicine**

"Persistent symptoms and clinical findings in adults with post-acute sequelae of COVID-19/post-COVID-19 syndrome in the second year after acute infection: population-based, nested case-control study"

Point-to-point responses

**Comments from the academic editor:**

**#1:** In general, the detail of descriptive data are noteworthy just to describe the patterns being seen. It is difficult to make much of the associations, as many are likely confounders or due to reverse causation (as the authors point out).

One recommendation I would make is to simply use association throughout the manuscript so readers do not conflate anything as causation (e.g., predictors doesn't seem like the best epidemiological description but is used frequently).

**Response:** We are aware of this important point and paid attention to use a terminology that does not imply causality. We now checked the terminology throughout the manuscript and used alternative wording (e.g. “associated with ….”. We note that the terms “predicts”/”pre-dictors” were only used for describing the association of baseline/phase 1 variables and (subsequent) case-control status changes at the clinical assessment phase 2. Of course, this does not imply causal relationship, but simply addresses a variable preceding the outcome of interest and improves the discrimination with respect to the outcome.

**#2:** I think also challenging to know what types of symptoms or vulnerabilities to symptoms may have existed prior to COVID even though groups are defined by reporting <80% recovery.

**Response:** As emphasized several times in the manuscript (e.g. page 25), only symptoms not already present before the acute infection were considered in the case definition. The prevalance of symptoms before (and during and after) the acute infection has been displayed in every detail in the previous EPILOC phase 1 paper **(see supplemental Figure B in Peter et al. BMJ 2022)**. Preexisting illness/disease was evaluated through questions on pre-existing conditions (and now again shown for the phase 2 participants; **see table S1 in the present mansuscript)** and on comorbidities **(see table 1 of the revised manuscript)**. In the earlier EPILOC phase 1 paper we analysed (and found) associations between pre-existing illness(es) and PCS development while in the present paper we concentrated on factors associated with persistence of PCS (not development) or recovery from PCS.

**#3:** It is quite interesting that there are substantial differences in the patient-reported outcomes but much less so in physiologic measurements.

**Response:** We totally agree. Symptoms, in fact, reportedly persisted in the majority of cases **(as summarized in the discussion on page 18)**, but laboratory tests (including inflammatory, endocrine and coagulation markers among others) or resting echocardiographic results were not different (or showed only subtle differences of uncertain clinical significance) between persistent cases and stable controls after adjustment for covariates and could not discriminate cases from controls **(summary also in discussion page 18)**. As we write in our results section **page 16** *„Notably, there was a statistically significant association for CRP, HbA1c, and D-dimers before adjustment for BMI and smoking (data not shown)*.“ Therefore, adequate adjustment for potential confounders is a critical issue in this kind of observational research – besides limiting selection bias.

**#4:** Another main recommendation would really to focus mostly on the descriptive nature of the findings (which the authors do an excellent job and provide unique ways of looking at the patterns in the data). I am not sure that these data really tell us too much about the pathophysiology of PASC (except perhaps some things that don't appear to be happening), but I think that is okay as the level of descriptive detail they are able to provide is noteworthy in and of itself.

**Response:** We agree, and would like to thank you for the appreciation of this primarily descriptive work, challenging somehow earlier work on putative pathophysiology and biomarkers. This large study that used comprehensive clinical evaluation needed to focus on the descriptive nature of the data and used them to support previous or generate new hypotheses rather than test specific ones. Adequate adjustment for confounding was a most important issue in these analyses **(see response to reviewer 3)** to avoid misinterpretation of patterns that may support false conclusions. The findings we present may be also useful to question and perhaps refine the so far suggested pathophysiological hypotheses in the field. **In the abstract** we now state: „…. our findings do not support viral persistence, EBV reactivation, adrenal insufficiency or increased complement turnover as pathophysiologically relevant for persistent PCS.“ **(see also discussion section, page 26)**. We also point out that the research on the pathophysiology of PCS that is clearly needed should – based on our findings – include the role of skeletal muscle metabolism as well as neurometabolic and neuroinflammatory disorders and dysautonomia **(see discussion, page 26/27)**.

**Comments from reviewer 1:**

**General:** This is a comprehensive study of the characteristics and predictors of Long COVID, and includes a large number of PROMs and objective measures. The introduction is relevant, methods are appropriate and the results are presented in a logical and clear manner. It is suitable for publication in its current format.

**Response:** Thank you very much for the very positive evaluation of our work and the recommendation for publication.

**#1:** It may be helpful to include the circulating variants of concern at the time this cohort was first established, as there is some evidence to suggest Long COVID risks differ across variants.

**Response:** We do agree and make now reference to our previous paper and again briefly summarize the relevant information . A very brief para was introduced **(see methods, page 6)**: *“* *We estimated that most participants were infected with the wild type of SARS-CoV-2, less than 15% of the cohort with B.1.1.7 (alpha) and less than 1% with B.1.351 (beta).”*

**#2:** The discussion, while relevant, is long. It may benefit from some editing down to distil the key points in a more succinct fashion.

**Response:** We slightly edited the discussion and shortened it **(see discussion section)**.

**#3:** It would also benefit from the authors giving some hypothesis as to why educational status is associated with Long COVID outcomes.

**Response** **(see also response to reviewer 2, #12)**: Finding educational level to be associated with PCS development has been reported in diverse contexts such as USA, UK and Africa. As we all know, educational status is strongly associated with many underlying social, economic, life style and behavioral factors that are related to many health-related outcomes. Smoking, unhealthy behaviors, unhealthy diet, and also increased body mass index (to name only a few) are all related to lower educational status – and also to increased risk of PCS. Furthermore, better education is often linked to a better job, a higher income and therefore access to resources such as healthier diet and better housing. Education improves health literacy and disease management. Psychological factors such as better coping with stress and disease, a hogher resilience in general, and better social networks may also be associated with health outcomes. As all these factors are difficult and time-consuming to assess in the context of an observational study, educational status is often used as proxy. Since we have no other specific hypotheses about that association we added a very brief text on this point in the discussion (to account for the anyway relatively long discussion section, see your #2) **(see page 19)**.

**#4:** Nevertheless, I don't think any of the above points should necessarily be a barrier to publication.

**Response:** Again, thank you very much for the very positive evaluation of our work and the recommendation for publication.

**Comments from reviewer 2:**

**#1:** In the Introduction section, it is stated that new symptoms were self-reported. The reliability of self-reported data might be discussed.

**Response:** We agree. The reliability of the self-reported data poses a certain limitation that is, however, prevalent in almost any PCS research.

We note that, in general, the reliability of symptom reporting in the acute phase of COVID-19 was described as moderate to good across most items, with inter-rater intra-class correlation estimates ranging from 0.50 to 0.90 (see Rofail et al. JAMA Network Open 2022; DOI: 10.1001/jamanetworkopen.2022.39053). A good reliability was also reported in a study which evaluated the psychometric properties in patients with PCS attending a post-COVID-19 rehabilitation clinic (see O`Connor et al. Journal of Medical Virology 2022; DOI: 10.1002/jmv.27415). Internal consistency of the overall COVID-19 Yorkshire Rehabilitation Scale was good (Cronbach's *α* = 0.891). It was used to record patients' symptoms, functioning, and disability. Individual subscales of self-reported symptoms also demonstrated good reliability. Deletion of the items noted to have poor scaling assumptions and targeting improved the reliability of the symptom severity subscale (swallowing, incontinence removed; Cronbach's *α* 0.79–0.81) and the additional symptoms subscale (fever, skin rash removed; Cronbach's *α* 0.70–0.74).

As additional remark, it was an important methodological component for the study team to attempt to increase validity by adding a number of established and validated questionnaires in addition to the evaluation of the patient reported symptoms. The symptoms were also graded in terms of severity and impairment. The combination of both approaches allowed a more valid estimation of severity **(see manuscript, page 13)** .

For the four main symptom clusters, we explicitly display the results of the “simple” symptom questions (yes/no/grade of impairment) together with the results of corresponding validated questionnaires for both cases and controls **in table 2** of the manuscript. We note some differences in the prevalence of symptoms versus ratings at the cut-offs, but these were minor in our view and did not change the case-control assessments.

**#2:** It might be clarified as to whether PCS recurrence after the study period is possible, for the controls.

**Response:** We agree and added a brief statement in th discussion section **(see page 26)** clarifying that PCS recurrence after the study period is possible: “There have been reports about undulating clinical manifestations of PCS, and there is certainly a continued risk of relapsing PCS both in improved cases as well as development of PCS in so far stable recovered controls. This aspect which, due to study design and methodology, could not be fully captured in this work needs additional research.”

**#3:** In the Introduction section, it is stated that "We hypothesized that roughly half of the cases following the invitation would be persistent cases..."; it might be clarified as to how this estimate was obtained.

**Response:** We now added in the text that this hypothesis was: “… based on estimates from experience in our and other PCS centers and the literature available at the time of planing this study ….”**(see page 6)**.

**#4:** In the Materials and methods section, it is stated that the study categorized 28.5% of respondents as PCS, and 38% as PCS-free. The status (and clinical implications) of the remaining 33.5% might be clarified, possibly together with the definitions in the following paragraph.

**Response**: We apologize for the confusion that might have arisen based on an incorrect definition of the controls in that part of the manuscript. “Subjects who had recovered to >80 […] qualified as controls” should read (and now reads, **see page 7**) “Subjects who had recovered to 100% […] and reported no new symptoms of grade moderate to strong qualified as controls”. We corrected the text accordingly and also clarified **(see page 7)** that “individuals qualifying as neither PCS cases nor controls were not invited.” This 33.5% include, for example, individuals who indicated moderate-severity symptoms, but reported general health to be 90-100% or individuals who reported impaired work ability and/or general health (≤80%) but only mild symptoms, etc.

**#5:** From Supplementary figure S1, a large proportion of cases declined or were unable to attend. Might this have biased the population for Phase 2? This might be discussed.

**Response:** Indeed, there might be some response bias. As shown and discussed in the manuscript, study participation was higher in cases than in controls from phase 1, and subjects with risk factors (e.g. smoking, obesity) were less likely to respond **(see page 25)**. Participation rates by different characteristics can be found in **supplemental figure S2 (which is now figure S1)**. It appears that factors like smoking and obesity similarly affected participation rates of cases and controls. For those invited but did not participate in phase 2, we asked for the reasons which were answered by 45.1% of cases and 44.4% of controls, respectively. The main reasons in cases (versus controls) were “lack of time” 58.1% (versus 67.2%), “distance to study centre” 14.0% (versus 12.5%), “poor health due to other diseases” 10.2% (versus 2.6%), “currently re-infected” 5.6% (versus 3.7%), “poor health due to Covid” 5.2% (0.4%), respectively. We now added this information to the **(new) figure 1 legend**.

**#6:** Next, it is stated that "The unequal sampling ratio was based on the assumption that a significant number of phase 1 cases might have had recovered until presentation in phase 2". Again, how was this particular sampling ratio derived?

**Response:** This sampling ratio was derived from (early) estimates as pointed out in the response to #5 (above).

**#7:** In the Data sources and measurements section, it is stated that vaccines received was part of the data. Was there any data on the type of vaccine?

**Response:** Yes, we have the type of vaccine recorded. We included it into the footnote of **(the new) supplementary table S1**.

**#8:** For the data used, was there a possibility of any missing data? If so, how were such cases handled (exclusion, imputation)? This might be clarified.

**Response:** Yes, there were missing data,.Unfortunately, not all laboratory and diagnostic tests could be completed for all cases and controls; there are also a few missings in patient-reported outcomes. Tables and figures contain the numbers evaluable. The missing observations were excluded in specific analyses. We now clarified the handling of missing data in the methods section **(see page 10)**.

**#9:** In the Symptoms and signs section, symptom clusters were discussed. It might be clarified as to how these clusters were formed - were they defined beforehand, or post-hoc?

**Response:** The symptom clusters were defined based on data from phase 1 which is explained in details in the phase 1 publication **(details see in Peter et al. BMJ 2022, ref. no. 32)** to which we refer - for reasons of space (and the necessity to shorten the current manuscript) **(see page 7** ).

For your information (as described in Peter et al 2022): “We used a two step approach to identify symptom clusters (not present before the SARS-CoV-2 infection). Firstly, we identified strongly correlated current symptoms (not present before the acute SARSCoV-2 infection) by using exploratory polychoric factor analysis (using the oblimin rotation) based on symptom severity (not present, no impairment, light impairment, moderate impairment, or strong impairment). To identify the ideal number of factors, we used “parallel” analysis. Secondly, we included each symptom into the cluster (identified in the first step as factor) for which its factor loading was highest.”

**#10:** In the Results section, differences between cases and controls were noted. It might then be discussed as to the choice of criteria used for matching to controls, and the matching algorithm details (e.g. how much of a difference was acceptable, was multiple matching allowed, etc.).

**Response:** Thanks for pointing out that the matching algorithm should be described in more detail. We performed exact frequency matching by sex and age-group. Our targeted sampling ratio was ten cases to six controls (see above), for example, for ten female cases of the age-group 40-49 years, we invited six female controls of the same age-group. We now added this detail about the matching procedure in the methods section **(see page 7)**.

**#11:** In the Risk for PCS persistence subsection, "most prominent" might be "most prominently".

**Response:** We changed this typo accordingly **(see page 11)** – thank you.

**#12:** Lower educational status appears a common risk factor - might this however be due to hidden factors, e.g. type of employment?

**Response:** (see also comments to reviewer 1 #3). We added text in the Discussion section on this point. Notably, employment status was – besides education a factor associated with cas-control status change **(see [new] suppl table S2)**. Educational status is strongly associated with many underlying social, economic, lifestyle and behavioral factors that are related to many health-related outcomes. In this particular case, it was a relevant factor independent of employment status in adjusted analysis. We admit, however, educational status is linked to a variety of variables that are, in turn, related to health issues - income, access to resources such as healthier food and better housing, health literacy, better coping with stress and disease, better social networks, etc. We used educational status as a kind of proxy. In Germany, there are socioeconomic indivators such as the UK indices of deprivation available.

**Comments from reviewer 3:**

**General:** This interesting article by Peter & Nieters and colleagues covers the clinically relevant and still timely topic of Post-COVID-Syndrome. Although the SARS-CoV-2 pandemic seems to be contained, a significant proportion of people still suffer from post-infectious symptoms. The strength of the present study is its population-based design and the detailed clinical follow-up including not only vast laboratory tests but also cardiopulmonary exercise-testing and other objective measurements. Many potential hypotheses for symptom persistence have been addressed including viral persistence, EBV reactivation, adrenal insufficiency, and increased complement turnover. The data of the current study did not show any results to support one of these hypotheses. The absence of any new evidence to further support these hypotheses maybe the most relevant news of this study. This could be highlighted in more detail in the abstract.

**Response** (see also response to academic editor comment #4): Thank you for the very positive evaluation of our work. We highlighted this in more detail **in the abstract** where we now state: „…. our findings do not support viral persistence, EBV reactivation, adrenal insufficiency or increased complement turnover as pathophysiologically relevant for persistent PCS.“ **(see also discussion section, page 26)**. We also point out that the research on the pathophysiology of PCS that is clearly needed should – based on our findings – include the role of skeletal muscle metabolism as well as neurometabolic and neuroinflammatory disorders and dysautonomia **(see discussion, page 26/27)**.

**#1:** Especially as the manuscript is rather lengthy and generally would benefit from some shortening, this essential message should not stay unrecognized for the readership. In addition, the relevance of the education level in absence of clear biomedical associations to the subjectively perceived symptoms leaves room to speculate on the pathogenesis of PCS. Based on their findings, authors maybe could include some speculations on the potential existence of different phenotypes of PCS that might be more or less related to biological vs. psycho-social aspects of each individual.

**Response:** We shortened the manuscript as recommended. We admit that during the work, we internally discussed a lot about more clinical symptom- or symptom cluster-based phenotyping of the cases. Due to the observations of large overlaps of the key symptom clusters and the dynamics observed (changes over time in the prevalence of symptoms/symptom clusters, here shown by the comparison between phase 1 and phase 2, **see suppl. figure S3**) we did not further focus on this aspect in the present work. We were more interested in phase 2 as a follow-up study investigating the prognostic aspects of the disease in more general terms (factor associated with improvement and time to freedom from symptoms and impaired daily functioning) – besides the cross-sectional analyses of medical tests. Also, we have already completed another follow-up study with clinical exmaonation and performed a number of ancillary studies evaluating the biological correlates (neurolesional biomarkers and imaging) of (persistent) cases (with more or less cognition deficits) and controls.

**#2 (minor comments):** For the generalizability of the results, the flow-chart should not be part of the supplement, but be included in the main manuscript. The frequency of 3,300 cases and 4,400 controls do not sum up to the EPILOC phase 1 cohort von 11,710. Could you please elaborate on that?

**Response:** We apologize for the confusion about the numbers (see also comments to reviewer 2 #4). There was an error describing the definition of controls “Subjects who had recovered to >80 […] qualified as controls” should (and now does) read “Subjects who had recovered to 100% […] qualified as controls”. We now corrected that **(see Methods section, page 7)** and clarified that individuals qualifying as neither PCS cases nor controls were not invited (the remaining 4000 individuals). We have now included the flow chart in the main manuscript **(see new figure 1)**.
