# Supplementary material for: Persistent symptoms and clinical findings in adults with post-acute sequelae of COVID-19/post-COVID-19 syndrome in the second year after acute infection: A population-based, nested case-control study
Source: PLoS Med. 2025 Jan 23;22(1):e1004511. doi: 10.1371/journal.pmed.1004511 (PMC12005676; doi:10.1371/journal.pmed.1004511)
Supplement: Attachment — Submitted filename: EPILOC Ph2_3rd REVISION_point-to-point responses.docx [file pmed.1004511.s025.docx]

**(PMEDICINE-D-24-01834R2) to PLOS Medicine**

"Persistent symptoms and clinical findings in adults with post-acute sequelae of COVID-19/post-COVID-19 syndrome in the second year after acute infection: population-based, nested case-control study"

Point-by-point responses

**Comments from Reviewers:**

**Reviewer #2:**

**1. More specific details on the hypothesis that half of the cases would be persistent might be stated, in particular the literature consulted, and any study-specific factors considered.**

As described earlier (and clarified in the previous revision), we based the assumption on (rough) estimates from experience in our and other (local) PCS centers and the literature available at the time of planning this study; the literature at at that time (second half/end of 2021), however, generally reported follow-up times of not more than 12 months (and often shorter periods of time). The papers published at that time by Nehme et al (Ann Intern Med 2021) and by Haverall et al (JAMA 2021) were the major source of (external) information considered by us to indicate a substantially reduced prevalence of complaints compataible with PCS between 6-12 months and 12-24 months after index infection. Together with the experience at the centers of approximately 50% of the long covid outpatients indicating to have improved health after several months, this was the basis of the eventual assumption. Since this was more a kind of extrapolation than solid projections we would prefer to leave a more detailed discussion of these points out of the manuscript.

**2. It is clarified that missing observations were excluded in specific analyses. Where possible, the proportion of missing data for each relevant variable might be stated.**

We would like to refer the reviewer to the tables and figures in which we indicated the number of evaluable subjects for the different investigation items. We hope this is making additional information in writingin tables or text about proportions missing unnecessary.

**3. From Supplementary table S2, two or more vaccine shots appears correlated with a lower odds ratio for cases improved, and a higher odds ratio for controls worsening (although the effect may not be significant). This observation might be briefly discussed if appropriate.**

This is correct, but the effect was not significant. Vaccination was discussed briefly (page 19, 2^nd^ para), but we did not elaborate that (statistically non-significant) tendency of a possible small impact of vaccination on non-improvement among case patients or even on worsening among controls. This would need, in our opinion, a few more sentences with discussion of a possible post-vac phenomenon which we would love to avoid in this paper.

**Reviewer #3:**

**I have no further comments.**

Thank you very much.

**Requests from Editors:**

1. **Data Availability Statement: Please provide instructions with contact information (web or email address) for obtaining the data. Please note that a study author cannot be the contact person for the data.**

done - the contact is: dauc.EPILOC@uni-ulm.de

**2. General: please ensure that you use patient centric language thoughout the paper. For example, you should avoid referring to individuals as ‘cases’ and ‘controls’ (throughout paper; eg, in the introduction “PCS patients” should be “patients with PCS”).**

done.

**3. Page 16, “...there was a statistically significant association for CRP, HbA1c, and D-dimers before adjustment for BMI and smoking (data not shown).” PLOS journals do not allow statements supported by "data not shown" or "unpublished results." For such statements, authors must provide supporting data (in the supplement) or cite public sources that include it.**

appropriate information added (p values for unadjusted analyses were already included in the last version).

**4. Abstract: Please combine the Methods and Findings sections into one section, per PLOS Medicine style.**

done.

**5. Abstract: Please spell out ME/CFS (also at first mention in the main text).**

done.

**6. Abstract: Please consider adding ‘relative to controls’ to the end of the following sentence, “In persistent cases, handgrip strength, maximal oxygen consumption, and ventilator efficiency were significantly reduced”.**

wording added as suggested.

**7. Abstract: Please spell out post-exertional malaise throughout, in order to minimize the use of abbreviations.**

done.

**8. Please revise the Author summary so that it conforms to PLOS Medicine style (headings and bullet points). The Author Summary should immediately follow the Abstract in your revised manuscript. This text is subject to editorial change and should be distinct from the scientific abstract. Ideally each sub-heading should contain 2-3 single sentence, concise bullet points containing the most salient points from your study. In the final bullet point of ‘What Do These Findings Mean?’, please include the main limitations of the study in non-technical language. Please see our author guidelines for more information: https://journals.plos.org/plosmedicine/s/revising-your-manuscript#loc-author-summary**

done.

**9. Results, p 10: Please insert the exact value where you state “circa 65%”**

done.

**10. Results, p 10, line 5: Please remove ‘respectively’ at the end of the sentence, as it is not necessary**

done.

**11. Results, p 13: It is unclear what you mean by “somehow overrated” and “somehow underrated” with regard to specific symptoms comparing self-report and validated questionnaires. Please consider revising the wording in the interest of clarity.**

deleted.

**12. The Supplementary text does not appear to have been carried over from the previous revision; the supplementary file includes only display items (tables, figures). Please provide the supplement as one continuous PDF file.**

reformatted and submitted as needed and requested.

**13. Acknowledgments section: Does the list provided in this section include patients? If so, do you have permission to disclose their names?**

no (named) patients were included here; now clarified in this section.

**14. Please ensure that the study is reported according to the STROBE (or appropriate STOBE extension) guideline (available from: https://www.equator-network.org/reporting-guidelines/strobe) and include the completed STROBE (or STROBE extension) checklist as Supporting Information. When completing the checklist, please use section and paragraph numbers, rather than page numbers. Please add the following statement, or similar, to the Methods: "This study is reported as per the Strengthening the Reporting of Observational Studies in Epidemiology (STROBE) guideline (S1 Checklist)." When completing the checklist, please use section and paragraph numbers, rather than page numbers**

done.
