# Supplementary material for: Persistent symptoms and clinical findings in adults with post-acute sequelae of COVID-19/post-COVID-19 syndrome in the second year after acute infection: A population-based, nested case-control study
Source: PLoS Med. 2025 Jan 23;22(1):e1004511. doi: 10.1371/journal.pmed.1004511 (PMC12005676; doi:10.1371/journal.pmed.1004511)
Supplement: Attachment — Submitted filename: EPILOC Ph2_4th REVISION_point-to-point responses.docx [file pmed.1004511.s026.docx]

**(PMEDICINE-D-24-01834R4) to PLOS Medicine**

"Persistent symptoms and clinical findings in adults with post-acute sequelae of COVID-19/post-COVID-19 syndrome in the second year after acute infection: population-based, nested case-control study"

Revision with point-to point responses/comments to requests from the Editors

**Please address the first two comments from Reviewer 2 by incorporating the suggested changes. We note that Nehme et al (Ann Intern Med 2021) and Haverall et al (JAMA 2021) were major sources considered when planning your study, yet are not cited in your reference list. A detailed explanation is not required; it would be satisfactory to include that hypotheses were based on the available literature (with citations) and from your clinical experience at the centres mentioned.**

done as requested

**In addition, it would be helpful to include % missing data in the relevant tables, as per the reviewer’s suggestion.**

We would like to refer the reviewer again to the tables and figures in all of which we indicated the number of evaluable subjects for the different investigation items. We see no reason to add missing numbers or percentages where the actual numbers of evaluable study participants are given.

**Please see below a list of further minor points requiring attention. The list below may appear lengthy, but most of these are more minor points which should not require a substantial amount of time to attend to.**

**General comments:**

**Please replace "subject" with participant, patient, individual, or person throughout the manuscript.**

done as requested

**Please use person-first language throughout e.g. patients with persistent PCS.**

done as requested

**Throughout the paper, please adapt reference call-outs to the following style: "... every year [1,2]." (noting the absence of spaces within the square brackets, and placement of the reference call-out preceding punctuation).**

done as requested

**Please include continuous line numbers in your revised manuscript.**

done as requested

**To help us extend the reach of your research, please provide any Twitter handle(s) that would be appropriate to tag, including your own, your co-authors’, your institution, funder, or lab.**

the twitter/𝕏 address of the corresponding author was now added in the author for correspondence section of the manuscript; other 𝕏 addresses of interest are:

@Uniklinik_Fr

@uniklinik_hd

@uni_tue

@uni_ulm

@mwk_bw

**Title: Please revise your title to: “Persistent symptoms and clinical findings in adults with post-acute sequelae of COVID-19/post-COVID-19 syndrome in the second year after acute infection: A population-based, nested case-control study”**

done as requested

**Abstract:**

**Please note that the word limit for Abstracts (500 words) can be slightly exceeded to allow the following changes to be incorporated. We suggest also presenting the most salient findings within the Abstract.**

done as requested (748 words)

**Please structure your abstract using the PLOS Medicine headings (Background, Methods and Findings, Conclusions).**

done as requested

**Abstract Background: Please provide some brief context of why the study is important.**

done as requested

**Please omit 'were' from "New symptoms or PCS development among control subjects was associated with were an intercurrent..."**

done as requested

**Please define all abbreviations at first use.**

done as requested

**Please ensure that all numbers presented in the abstract are present and identical to numbers presented in the main manuscript text.**

checked as requested

**Please provide brief demographic details of the study population (e.g. sex, age, ethnicity, etc)**

done as requested

**Please quantify the main results (with 95% CIs and p values).**

done as requested (and as far as space allowed)

**Please provide the range for length of time between phases 1 and 2, in addition to the median.**

done as requested

**Please include the important dependent variables that are adjusted for in the analyses.**

now listed as requested

**In the last sentence of the Abstract Methods and Findings section, please describe 2-3 of to the best of our knowledge’ the main limitations of the study's methodology.**

done as requested

**Please begin your Abstract Conclusions with "In this study, we observed ..." or similar, to summarize the main findings from your study, without overstating your conclusions. Please emphasize what is new and address the implications of your study, being careful to avoid assertions of primacy.**

done as requested

**Author Summary:**

**Thank you for providing an Author Summary. Please temper assertions of primacy by adding ‘****to the best of our knowledge’ or similar to the sentence “The long-term prognosis of this post-COVID-19 syndrome (PCS) is unknown.”**

We do not think that this is required at this statement, but admit that it may be quite adequate for the subsequent sentence “evidence of measurable single or multiple organ dysfunction and pathology and their correlation with self-reported symptoms in patients with non-recovery from PCS for more than a year have not been well described” – so we included the “to the best od our knowledge” here

**Please revise “...adjustment for possible confounders….” to include a couple of examples.**

done as requested

**Please revise to “...but we did not identify major pathology in laboratory investigations.”**

done as requested

**Methods:**

**Did your study have a** **prospective protocol or analysis plan? Please state this - either way - early in the Methods section. If a prospective analysis plan (from your funding proposal, IRB or other ethics committee submission, study protocol, or other planning document written before analyzing the data) was used in designing the study, please include the relevant prospectively written document with your revised manuscript as a Supporting Information file to be published alongside your study, and cite it in the Methods section. A legend for this file should be included at the end of your manuscript. If no such document exists, please make sure that the Methods section transparently describes when analyses were planned, and if/when reported analyses differed from those that were planned. Changes in the analysis-- including those made in response to peer review comments-- should be identified as such in the Methods section of the paper, with rationale. If a reported analysis was performed based on an interesting but unanticipated pattern in the data, please be clear that the analysis was data-driven.**

the study handbook is only available in German language; we now added a short paragraph in this issue clarifying that all investigations and analyses were done as initially planned except some virological analyses (the PCR analysis of stoll samples and spike protein antigen assays) that were stopped after more than 100 negative results in samples from patients with persistent PCS (now briefly mentioned in the Methods section)

**Please provide a copy of the original symptom questionnaire as a supplementary file. We do not require copies of validated questionnaires.**

done as requested (S1 Appendix)

**Please define ACTH and DHEA-S at first use.**

done as requested

**Please add the following statement, or similar, to the Methods: "****This study is reported as per the Strengthening the Reporting of Observational Studies in Epidemiology (STROBE) guideline (S1 Checklist)."**

done as requested

**Results:**

**Page 11 - Please revise to “...factors associated with improvement (either to intermediate or control status)...”.**

done as requested

**Page 12 - Please revise to “...or as adjusted average ratings (figure 3).”**

done as requested

**Page 12 - Please remove ‘somehow’ from “...symptom cluster varied somehow…“.**

done as requested

**Page 13 - Please remove ‘somehow’ from “..the frequency of individual symptoms (of any degree) among case patients with PEM (lasting >14 hours) differed somehow from those who had no PEM”**

done as requested

**Page 14 - please avoid use of causative language; please revise to “...including the perceived impact of any illnesses or adverse condition …”**

done as requested

**Page 14 - Please revise to “...grip strength was lower in women than men (30.9kg versus 50.7 kg)...”.**

done as requested

**Page 14 - Please revise “...(adjusted systolic mean: 80 to 81 mmHg for all subgroups, p=0.88; diastolic mean: 128 to 131 mmHg for all subgroups, p=0.21).“ - please clarify values for SBP and DBP.**

sorry – now corrected (many thanks)

**Page 14 - For paragraph beginning “Differences were observed for FEV1 and FVC, SpO2 at rest, and several CPET derived variables”, please clarify in which table results are provided, as this does not appear to be Table S4 as currently stated. Please quantify results with p values and 95% CIs, at least in the tables.**

done as requested

**Discussion:**

**Please present and organize the Discussion as follows: a short, clear summary of the article's findings; what the study adds to existing research and where and why the results may differ from previous research; strengths and limitations of the study; implications and next steps for research, clinical practice, and/or public policy; one-paragraph conclusion.**

done as requested

**Please remove all subheadings within your Discussion e.g. Trajectories.**

done as requested

**Please remove the information on competing interests and funding from the end of the main text. In the event of publication, this information will appear in the article metadata, via entries in the submission form.**

done as requested

**Page 19 - please revise ‘vaccine shots ‘ to ‘vaccine doses’.**

done as requested (including in corresponding tables)

**Figures:**

**Please consider avoiding the use of red and green in order to make your figure more accessible to those with colour blindness.**

redrawn as requested

**Please define all abbreviations used in the figure legend of each figure.**

done as requested

**Please clarify what is represented by the numbers within brackets in Figures 3 and S5, and add to the figure legend.**

done as requested

**Please indicate in the figure caption the meaning of the bars in Figure S6 and S8.**

explained (96% CI) as requested

**Tables:**

**Please define all abbreviations used in the table legend of each table.**

done as requested

**Please provide the unadjusted comparisons as well as the adjusted comparisons in Tables S2, S4.**

done as requested

**Where adjusted analyses are presented, please specify the variables controlled for in the table legend.**

done as requested

**Please clarify what is represented by the numbers within brackets in the tables e.g. %.**

done as requested

**When a p value is given, please specify the statistical test used to determine it in the figure legend.**

done as requested

**References:**

**Please ensure that journal name abbreviations match those found in the National Center for Biotechnology Information (NCBI) databases (http://www.ncbi.nlm.nih.gov/nlmcatalog/journals), and are appropriately formatted and capitalised. The first six authors should be listed prior to ‘et al’.**

done as requested

**Please also see https://journals.plos.org/plosmedicine/s/submission-guidelines#loc-references for further details on reference formatting.**

thank you

**Supplementary files:**

**Please note that supplementary figures S10 to S20 are not provided, but are referred to in the main text. Use whole numbers when naming your supporting information files. Combine separate parts (e.g., S11A and S11B) into one file (e.g. S11 Figure) or rename with whole numbers (e.g., Figure S11, Figure S12, and so on).**

done as requested

**Additional requests per Email (24-10-2024): Further to my previous email, please provide the unadjusted comparisons as well as the adjusted comparisons in Tables S2, S4, S5, S6, S7.**

done as requested
